# The efficacy and safety of biologics for patients with severe asthma: an umbrella review of systematic reviews and meta-analyses

**DOI:** 10.3389/fmed.2025.1573596

**Published:** 2025-05-30

**Authors:** Qionghua Xiao, Yuanming Huang, Bingyu Xue, Minghang Wang

**Affiliations:** ^1^National Regional TCM (Pulmonary Disease) Diagnostic and Treatment Center, the First Affiliated Hospital of Henan University of Chinese Medicine, Zhengzhou, China; ^2^The First Clinical Medical School, Henan University of Chinese Medicine, Zhengzhou, China

**Keywords:** biologics, severe asthma, efficacy, safety, umbrella review

## Abstract

**Introduction:**

Many systematic reviews and meta-analyses (SR/MAs) have evaluated the efficacy of biologic therapy for severe asthma. However, the quality of these SR/MAs is unclear, which may influence the selection of biologics and lead to misleading clinical decisions. Therefore, this umbrella review aims to objectively evaluate the quality of these SR/MAs and reassess the efficacy of biologic therapy for severe asthma.

**Methods:**

A systematic search was performed in PubMed, EMBASE, Cochrane Library, Web of Science, and MEDLINE databases. Literature screening and data extraction were conducted according to inclusion and exclusion criteria. Then, we evaluated the methodological quality of these SR/MAs using A MeaSurement Tool to Assess Reviews 2 (AMSTAR 2). In addition, the re-meta-analysis of study outcomes was performed applying R 4.3.3 software.

**Results:**

The umbrella review included 23 SR/MAs. In the evaluation of methodological quality, five SR/MAs were rated as high quality, one was rated as moderate, and 17 were rated as low or critically low. In terms of efficacy evaluation, biologics were associated with a 45% reduction in AER (RR: 0.55; *P* < 0.0001), a 57% reduction of asthma-related hospitalizations (RR: 0.43; *P* < 0.0001), an increase in the forced expiratory volume in 1 s (FEV1) of 0.13 L (*P* < 0.0001), a reduction in asthma control questionnaire (ACQ) scores by 0.33 points (*P* < 0.0001), an increase in asthma quality of life questionnaire (AQLQ) scores by 0.26 points (*P* < 0.0001), and a reduction in fractional exhaled nitric oxide (FeNO) levels by 22.52 ppb (*P* < 0.0001). In terms of safety evaluation, overall, biologics demonstrated favorable safety.

**Conclusion:**

This umbrella review has demonstrated that biologics have good efficacy and acceptable safety in the treatment of severe asthma. However, the methodological quality of included SR/MAs was mostly low or critically low, suggesting that we need to be cautious when interpreting the results of this study. Therefore, more high-quality SR/MAs are needed to provide robust clinical evidence.

**Systematic review registration:**

https://www.crd.york.ac.uk/PROSPERO/, identifier CRD42024607393.

## 1 Introduction

Asthma is a serious global health problem, affecting about 300 million people worldwide and causing about 250,000 deaths annually (1). What’s worse, patients with severe asthma experience a heavy burden of symptoms, exacerbations, and medication side effects, which may interfere with their daily life, sleep, and physical activity (2). Moreover, severe asthma leads to very high medical costs (3), which pose a major challenge in clinical practice.

The emergence of biologics provides more precise and effective treatment options for patients with severe asthma. Biologics can block the immuno-inflammatory cascade in the pathological course of severe asthma by precisely targeting specific inflammatory cytokines and their receptors (4). It mainly includes anti-immunoglobulin E (anti-IgE) treatment (omalizumab), anti-interleukin-5/5Rα (anti-IL5/5Rα) treatment (mepolizumab, reslizumab, benralizumab), anti-interleukin-4Rα (anti-IL4Rα) treatment (dupilumab), and anti-thymic stromal lymphopoietin (anti-TSLP) treatment (tezepelumab) (5). In previous systematic reviews and meta-analyses (SR/MAs), biologics have been shown to be beneficial for severe asthma (6–8). However, study results were not entirely consistent among different SR/MAs. Meanwhile, the unclear quality of these SR/MAs may affect clinical decisions. Umbrella reviews represent the pinnacle of evidence-based medicine, which can assess the quality of SR/MAs and systematically synthesize their findings (9). Thus, they could provide new insights for clinical practice.

In this umbrella review, the methodological quality of included SR/MAs was evaluated through applying A MeaSurement Tool to Assess Reviews 2 (AMSTAR 2). Moreover, we reassessed the overall efficacy and safety of biologics for patients with severe asthma. Meanwhile, we also evaluated the study outcomes of different types of biologics. Ultimately, this study aims to provide evidence-based support for the application of biologics in severe asthma, thereby facilitating precise treatment.

## 2 Methods

### 2.1 Study registration

The protocol of this study was registered in PROSPERO (Registration number: CRD42024607393). It was reported according to Preferred Reporting Items for Systematic Review and Meta-Analysis (PRISMA) statement (10). The detailed PRISMA checklist can be found in Supplementary Table S1.

### 2.2 Search strategy

Two authors (QX and BX) independently carried out the retrieval of literature. PubMed, EMBASE, Cochrane Library, Web of Science, and MEDLINE databases were searched for literature. We also reviewed the conference proceedings. The searched period ran from the date of establishment of databases until December 10, 2024. The search terms were as follows: “Mepolizumab,” “Reslizumab,” “Benralizumab,” “Omalizumab,” “Dupilumab,” “Tezepelumab,” “Asthma,” “systematic review,” and “meta-analysis.” The full search strategy was provided in Supplementary Table S2.

### 2.3 Study selection

After duplicate removal, two reviewers (QX and YH) individually examined the titles and abstracts of eligible articles that meet the inclusion and exclusion criteria, and excluded irrelevant studies. EndNote 20 software was applied to generate citations and remove duplicate articles (11). Then, two authors (QX and YH) independently reviewed the full texts of remaining articles and determined the final SR/MAs included in the umbrella review. All disagreements were resolved by the third independent author (MW).

### 2.4 Eligibility criteria

The literature included in this umbrella review met the following inclusion criteria. (1) Study design: This study only included eligible SR/MAs for analysis. (2) Participants: This umbrella review considered SR/MAs that focus on participants over 6 years old with severe asthma. (3) Intervention: The interventions of this study included biologic therapy with/without routine therapy. Currently available asthma biologics are as follows: anti-IgE treatment (omalizumab), anti-IL5/5Rα treatment (mepolizumab, reslizumab, benralizumab), anti-IL4Rα treatment (dupilumab), and anti-TSLP treatment (tezepelumab) (5). (4) Comparison: The control group received routine therapy or corresponding placebo. (5) Outcome: The literature was required to report 1 or more of the following outcomes: annualized asthma exacerbation rate (AER), the change from baseline in forced expiratory volume in 1 s (FEV1), asthma control questionnaire (ACQ) scores, asthma quality of life questionnaire (AQLQ) scores, asthma-related hospitalizations, blood eosinophils level, and fractional exhaled nitric oxide (FeNO) level. Moreover, we collected the data of adverse events (AEs) and severe adverse events (SAEs). Thus, we can evaluate the safety of biologics on patients with severe asthma.

The exclusion criteria of this umbrella review were as follows. (1) Articles for which the full text is not available, (2) Articles without available data, (3) Duplicate or retracted studies, (4) Articles in a language other than English, (5) SR/MAs of non-randomized controlled trials (RCTs), (6) Network meta-analysis.

### 2.5 Data extraction and quality assessment

Data extraction was conducted by two researchers (BX and YH). The extracted information of reviews included name of first author, year of publication, title of SR/MAs, database searched, number of RCTs, sample size per group, age per group, gender ratio per group, type and dose of biologics, treatment duration, type of comparisons, type of effect sizes, as well as effect sizes for efficacy and safety outcomes. Any discrepancies were resolved by discussion.

Two reviewers (QX and YH) independently assessed the methodological quality of included SR/MAs using the AMSTAR 2 tool (12). It includes 16 items, with 7 key items. The AMSTAR 2’s development team recommended focusing on the methodological conditions of key items and giving an overall evaluation. Methodological quality of each SR/MA was categorized as high, moderate, low, and critically low. All discrepancies were resolved by the third independent author (MW).

### 2.6 Statistical analysis

All analyses were conducted by “meta” package in R 4.3.3 software (13). Firstly, outcomes were expressed as risk ratio (RR) and mean difference (MD) with corresponding 95% confidence intervals (CIs). If the effect sizes in the SR/MAs were not MD or RR, we calculated the original data in the literature into these two types of effect sizes. Then, we evaluated the heterogeneity of included SR/MAs by using the Cochrane’s Q test and *I*^2^ statistics. *P* < 0.1 or *I*^2^ > 50% indicates significant heterogeneity, and the random-effects model was used (14). Otherwise, we chose fixed-effects model. Next, we calculated pooled RRs or MDs with 95% CIs for each outcome of different biologics. The results were presented clearly by texts, tables, and figures. *P* < 0.05 indicates statistically significant.

In addition, we conducted the sensitivity analysis to evaluate the influence of each study on overall outcomes.

## 3 Results

### 3.1 Study selection

**FIGURE 1:**
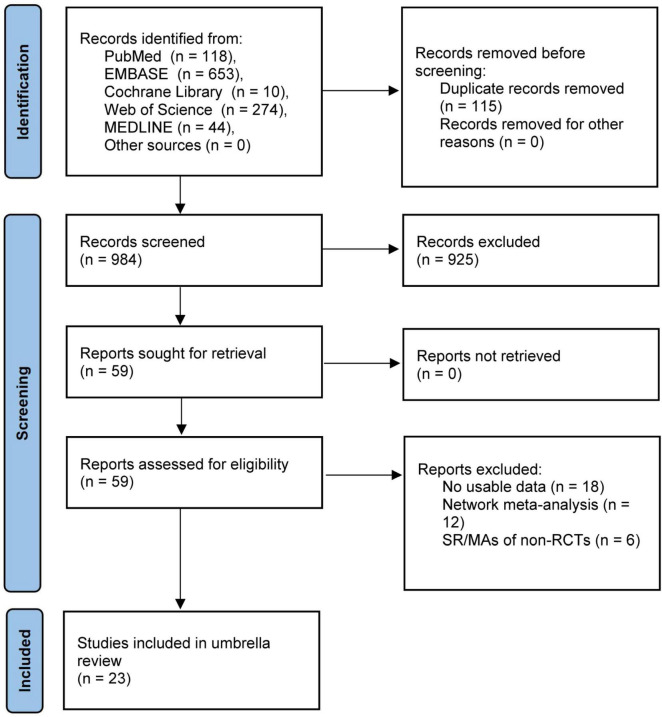
Flow chart diagram of study selection.

The flow chart of study selection was illustrated in Figure 1. A total of 1,099 SR/MAs were identified through searching PubMed, EMBASE, Cochrane Library, Web of Science, and MEDLINE databases up to December 10, 2024. After removing duplicate studies, filtering titles and abstracts, and reviewing the full texts, 23 studies with 112,513 patients were ultimately included in this umbrella review (6–8, 15–34). A full list of the excluded studies is provided in Supplementary Table S3.

### 3.2 Study characteristics

The characteristics of 23 included SR/MAs were summarized in Table 1. In terms of year of publication, all included SR/MAs were published between 2014 and 2024, all primary studies in SR/MAs were published between 1997 and 2022. Among the 23 SR/MAs, six focused on anti-IgE treatment, seven on anti-IL5/5Rα treatment, three on anti-IL4Rα treatment, three on anti-TSLP treatment, and four on multiple types of biologics. Treatment duration ranged from 1 to 60 weeks. All included SR/MAs compared biologics with placebo/standard of care.

**TABLE 1:** The study characteristics of included studies.

Author (year)	Number of primary studies (total sample size)	Period covered	Mean age/age range (year)	Gender ratio (female)	Type of biologics	Treatment duration	Outcomes
Tian et al. (15)	7 (*N* = 2,321)	2013–2017	48.35	1,481 (63.81%)	Benralizumab	NA	(1)(2)(3)(4)(8)
Liu et al. (16)	5 (*N* = 1,951)	2013–2016	12–75	NA	Benralizumab	Varied (8–56 weeks)	(1)(2)(3)(4)(8)
Liu et al. (17)	8 (*N* = 3,788)	2013–2017	12–75	2,215 (58.47%)	Benralizumab	Varied (1–56 weeks)	(8)(9)
Xiong et al. (18)	5 (*N* = 3,369)	2013–2018	NA	NA	Dupilumab	Varied (12–52 weeks)	(1)(2)(3)(4)(7)(8)
Zayed et al. (19)	4 (*N* = 2,992)	2013–2018	48.04 ± 14.59	1,866 (63.86%)	Dupilumab	NA	(1)(2)(8)(9)
Li et al. (20)	24 (*N* = 10,171)	2011–2022	6–82	6,238 (61.33%)	Anti-IL-5 treatment	NA	(8)(9)
Yancey et al. (7)	4 (*N* = 1,388)	2009–2014	12–82	NA	Mepolizumab	at least 24 weeks	(5)
Liao et al. (6)	11 (*N* = 3,578)	2001–2022	6–75	NA	Omalizumab	Varied (16–60 weeks)	(2)
Fu et al. (21)	3 (*N* = 1,380)	2001–2011	6–20	NA	Omalizumab	Varied (24–60 weeks)	(1)(8)(9)
Lai et al. (22)	6 (*N* = 2,749)	2002–2011	33.02	1,323 (48.13%)	Omalizumab	at least 52 weeks	(1)(8)(9)
Li et al. (23)	7 (*N* = 2,682)	2002–2021	6–17	NA	Omalizumab	Varied (16–52 weeks)	(1)(8)(9)
Rodrigo et al. (24)	3 (*N* = 1,381)	2001–2011	6–20	516 (37.36%)	Omalizumab	NA	(1)(5)(8)(9)
Li et al. (8)	4 (*N* = 1,366)	2011–2016	12–75	NA	Reslizumab	Varied (15–16 weeks)	(1)(2)(3)(6)
Lee et al. (25)	17 (*N* = 11800)	2009–2019	NA	7,236 (61.32%)	Benralizumab, Dupilumab, Mepolizumab, Reslizumab	NA	(1)
Agache et al. (26)	28 (*N* = 11,619)	2011–2019	6–75	NA	Benralizumab, Dupilumab, Mepolizumab, Omalizumab Reslizumab	Varied (12–56 weeks)	(1)(2)(3)(4)(5) (7)(8)(9)
Agache et al. (27)	37 (*N* = 11,138)	2011–2018	6–75	NA	Benralizumab, Dupilumab, Omalizumab	Varied (12–56 weeks)	(1)(2)(3)(4)(8)(9)
Agache et al. (28)	3 (*N* = 2735)	2016–2018	48.17 ± 14.50	NA	Dupilumab	Varied (24–52 weeks)	(1)(2)(3)(4) (7)(8)(9)
Chagas et al. (29)	3 (*N* = 1484)	2017–2022	50.42 ± 15.1	977 (65.84%)	Tezepelumab	Varied (48–52 weeks)	(1)(2)(3)(4)(6) (7)(8)(9)
Kyriakopoulos et al. (30)	48 (*N* = 16,350)	1999–2022	11–81	NA	Omalizumab, Mepolizumab, Reslizumab, Benralizumab, Dupilumab, Tezepelumab	NA	(1)(2)(3)(4) (5)(8)(9)
Lin et al. (31)	4 (*N* = 1,600)	2017–2021	12–80	1,012 (63.25%)	Tezepelumab	Varied (48–52 weeks)	(1)(2)(3)(8)(9)
Abdelgalil et al. (32)	4 (*N* = 1,875)	2017–2022	50.44 ± 14.63	1,192 (63.57%)	Tezepelumab	NA	(1)(2)(3)(4)(6) (7)(8)(9)
Normansell et al. (33)	25 (*N* = 6,382)	1997–2012	6–75	NA	Omalizumab	Varied (8–60 weeks)	(1)(2)(4)(5) (8)(9)
Farne et al. (34)	16 (*N* = 8,414)	2009–2022	NA	NA	Benralizumab, Mepolizumab, Reslizumab	Varied (16–56 weeks)	(2)(3)(4)(9)

NA, not available; Outcomes: (1) annualized asthma exacerbation rate, (2) the change from baseline in pre-bronchodilator forced expiratory volume in 1 s, (3) asthma control questionnaire, (4) asthma quality of life questionnaire, (5) number of hospitalizations due to asthma, (6) number of blood eosinophils, (7) fractional exhaled nitric oxide, (8) adverse events, (9) severe adverse events.

### 3.3 Quality assessment of included studies

The results of the methodological quality assessment of each SR/MA were presented in Table 2. The evaluation details of each SR/MA, the content of each item, and the overall quality assessment criteria were provided in Supplementary Table S4. Five SR/MAs were rated as high quality, one as moderate quality, thirteen as low quality, and four as critically low quality. Some of the key challenges included no list of excluded literature and reasons for exclusion (*n* = 17), no funding sources for the primary studies included in SR/MAs (*n* = 15), no provision of potential conflicts of interest (*n* = 5), and no investigation of the publication bias (*n* = 4).

**TABLE 2:** Methodological quality assessment of included studies using AMSTAR 2.

Author (year)	Quality level			
	High	Moderate	Low	Critically low
Tian et al. (15)			√	
Liu et al. (16)			√	
Liu et al. (17)			√	
Xiong et al. (18)			√	
Zayed et al. (19)			√	
Li et al. (20)			√	
Yancey et al. (7)			√	
Liao et al. (6)				√
Fu et al. (21)			√	
Lai et al. (22)		√		
Li et al. (23)			√	
Rodrigo et al. (24)				√
Li et al. (8)			√	
Lee et al. (25)			√	
Agache et al. (26)	√			
Agache et al. (27)	√			
Agache et al. (28)	√			
Chagas et al. (29)				√
Kyriakopoulos et al. (30)			√	
Lin et al. (31)				√
Abdelgalil et al. (32)			√	
Normansell et al. (33)	√			
Farne et al. (34)	√			

### 3.4 Overall evaluation of biologics

**FIGURE 2:**
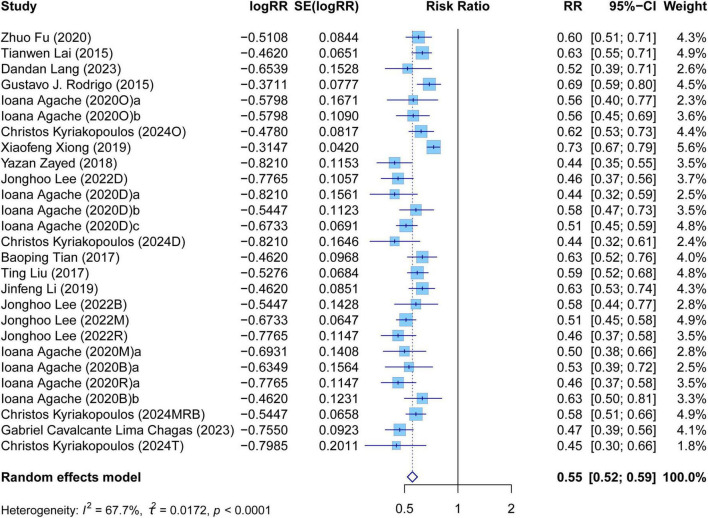
Forest plot of asthma exacerbation rate comparing biologics and controls.

**FIGURE 3:**
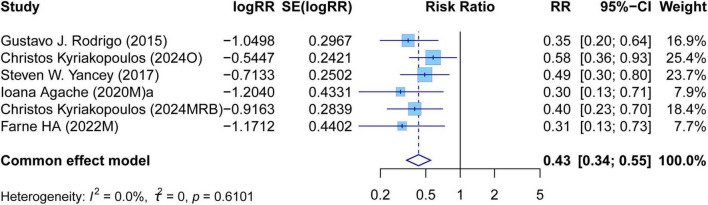
Forest plot of asthma-related hospitalizations comparing biologics and controls.

**FIGURE 4:**
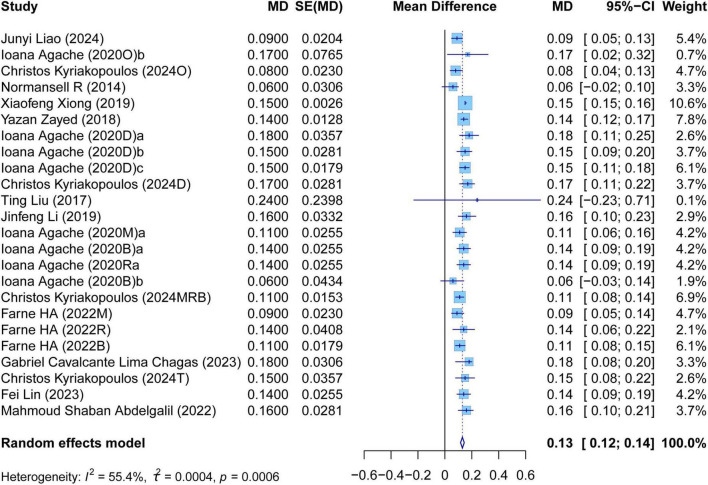
Forest plot of the mean change difference in forced expiratory volume in 1 s comparing biologics and controls.

**FIGURE 5:**
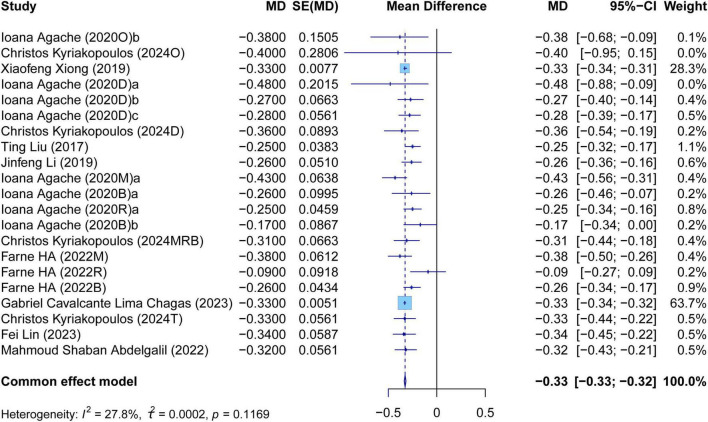
Forest plot of the mean change difference in asthma control questionnaire scores comparing biologics and controls.

**FIGURE 6:**
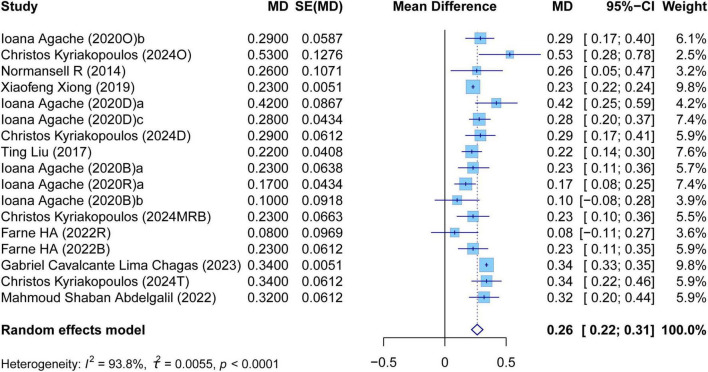
Forest plot of the mean change difference in asthma quality of life questionnaire scores comparing biologics and controls.

**FIGURE 7:**
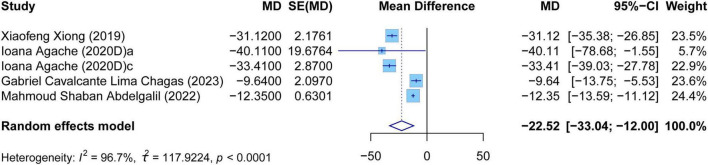
Forest plot of the mean change difference in fractional exhaled nitric oxide comparing biologics and controls.

**FIGURE 8:**
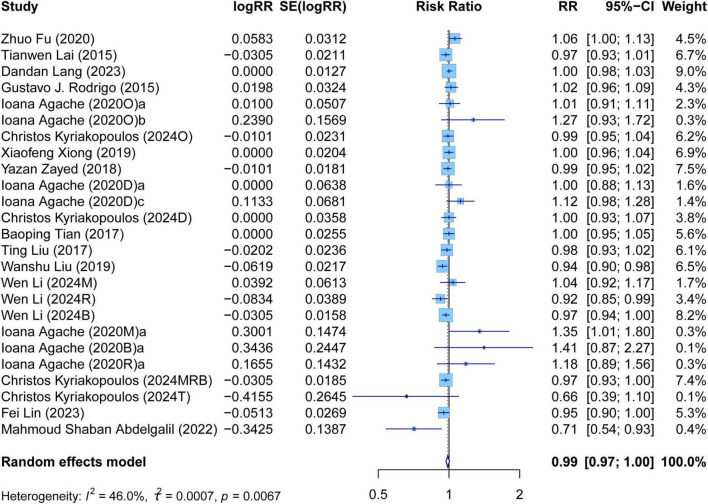
Forest plot of adverse events comparing biologics and controls.

**FIGURE 9:**
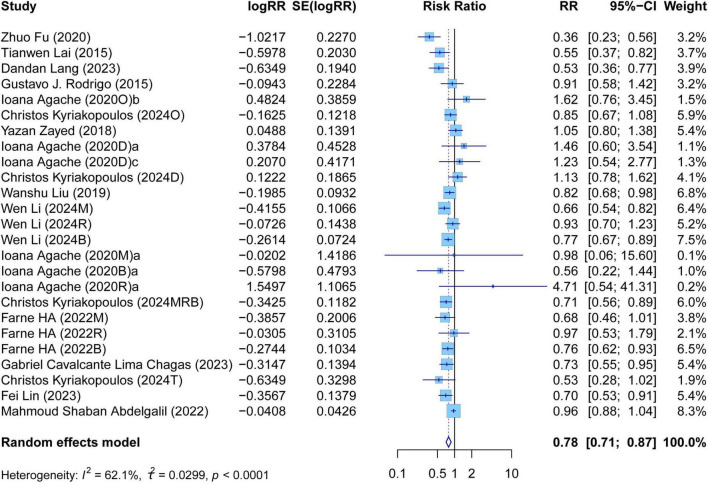
Forest plot of serious adverse events comparing biologics and controls.

The meta-analysis comprehensively assessed the efficacy and safety of biologics compared to controls across various outcomes, including AER, asthma-related hospitalizations, FEV1, ACQ scores, AQLQ scores, FENO levels, AEs, and SAEs. In terms of efficacy, biologics significantly reduced AER compared to the control group (RR: 0.55, 95% CI: 0.52–0.59, *P* < 0.0001, *I*^2^ = 67.7%) (Figure 2) and also led to a significant decrease in asthma-related hospitalizations (RR: 0.43, 95% CI: 0.34–0.55, *P* < 0.0001, *I*^2^ = 0%) (Figure 3). Regarding lung function, the FEV1 increased significantly in the biologics group (MD: 0.13, 95% CI: 0.12–0.14, *P* < 0.0001, *I*^2^ = 55.4%) (Figure 4). For patient-reported outcomes (PROs), ACQ scores demonstrated a significant reduction (MD: −0.33, 95% CI: −0.33 to −0.32, *P* < 0.0001, *I*^2^ = 27.8%) (Figure 5), while AQLQ scores exhibited a significant increase (MD: 0.26, 95% CI: 0.22–0.31, *P* < 0.0001, *I*^2^ = 93.8%) (Figure 6). Additionally, FeNO levels decreased significantly with biologics (MD: −22.52, 95% CI: −33.04 to −12.00, *P* < 0.0001, *I*^2^ = 96.7%) (Figure 7). In terms of safety, there was no significant difference in the risk of AEs between biologics and placebo (RR: 0.99, 95% CI: 0.97–1.00, *P* = 0.1591, *I*^2^ = 46%) (Figure 8). However, biologics significantly reduced the risk of SAEs compared to placebo (RR: 0.78, 95% CI: 0.71–0.87, *P* < 0.0001, *I*^2^ = 62.1%) (Figure 9).

There was high heterogeneity in AER, FEV1, AQLQ scores, FENO levels, AEs, and SAEs. It illustrated that these outcomes of biologic therapy for severe asthma vary greatly among different SR/MAs. In contrast, the heterogeneity of other outcomes was relatively low. It suggested that the results of each SR/MA may have a consistent trend.

### 3.5 Evaluation of different types of biologics

#### 3.5.1 Anti-IgE treatment

The meta-analysis evaluated the efficacy and safety of anti-IgE group compared with the control group across various outcomes, such as AER, asthma-related hospitalizations, FEV1, ACQ scores, AQLQ scores, AEs, and SAEs. In terms of efficacy, the anti-IgE group was associated with a significant reduction in AER (RR: 0.62, 95% CI: 0.58–0.66, *P* < 0.0001, *I*^2^ = 0%) (Supplementary Figure S1A). It also significantly lowered asthma-related hospitalizations in the anti-IgE group (RR: 0.47, 95% CI: 0.33–0.68, *P* < 0.0001, *I*^2^ = 42.5%) (Supplementary Figure S1B). Concerning lung function, FEV1 values showed a significant improvement in the anti-IgE group compared with the control group (MD: 0.08, 95% CI: 0.06–0.11, *P* < 0.0001, *I*^2^ = 0%) (Supplementary Figure S2A). Moreover, the assessment of PROs revealed that the anti-IgE group led to a significant decrease in ACQ scores (MD: −0.38, 95% CI: −0.64 to −0.12, *P* = 0.0037, *I*^2^ = 0%) (Supplementary Figure S2B) and a significant rise in AQLQ scores (MD: 0.32, 95% CI: 0.22–0.41, *P* < 0.0001, *I*^2^ = 39.1%) (Supplementary Figure S2C). In terms of safety, there was no significant difference in AEs between the two groups (RR: 1.00, 95% CI: 0.98–1.02, *P* = 0.8074, *I*^2^ = 29.5%) (Supplementary Figure S1C). Notably, fewer SAEs were observed in the anti-IgE group, with a statistically significant difference (RR: 0.68, 95% CI: 0.49–0.95, *P* = 0.0248, *I*^2^ = 75.7%) (Supplementary Figure S1D).

We observed high heterogeneity in SAEs. As illustrated in the forest plots, the SAEs reported from the first three authors trended to the left side of the axis, whereas the confidence intervals of SAEs from other authors intersected with “1.” It indicated that the incidence of SAEs fluctuates across different SR/MAs.

#### 3.5.2 Anti-IL5/5Rα treatment

The meta-analysis assessed the efficacy and safety of anti-IL5/5Rα group compared with control group through a range of outcomes, including AER, asthma-related hospitalizations, FEV1, ACQ, AQLQ, AEs, and SAEs. In terms of efficacy, the anti-IL5/5Rα group demonstrated a significant reduction in AER (RR: 0.56, 95% CI: 0.53–0.59, *P* < 0.0001, *I*^2^ = 28.4%) (Supplementary Figure S3A) and a significant decrease in asthma-related hospitalizations (RR: 0.40, 95% CI: 0.30–0.55, *P* < 0.0001, *I*^2^ = 0%) (Supplementary Figure S3B). Regarding lung function, FEV1 values improved significantly in the anti-IL5/5Rα group (MD: 0.12, 95% CI: 0.10–0.13, *P* < 0.0001, *I*^2^ = 0%) (Supplementary Figure S4A). Furthermore, the analysis of PROs showed that ACQ scores decreased significantly (MD: −0.27, 95% CI: −0.32 to −0.23, *P* < 0.0001, *I*^2^ = 42.3%) (Supplementary Figure S4B), and AQLQ scores increased significantly (MD: 0.20, 95% CI: 0.15–0.24, *P* < 0.0001, *I*^2^ = 0%) (Supplementary Figure S4C) in the anti-IL5/5Rα group compared to the control group. In terms of safety, there was no significant difference in the risk of AEs between the two groups (RR: 0.97, 95% CI: 0.95–1.00, *P* = 0.0591, *I*^2^ = 44.0%) (Supplementary Figure S3C). However, in the anti-IL5/5Rα group, the occurrence of SAEs was significantly lower than in the control group (Supplementary Figure S3D).

High heterogeneity was detected in ACQ scores and AEs, which indicated that these two outcomes vary significantly across different reviews and suggested further exploration of the reasons. Conversely, the low heterogeneity of other outcomes illustrated a certain degree of consistency among the included SR/MAs.

#### 3.5.3 Anti-IL4Rα treatment

The meta-analysis evaluated the efficacy and safety of anti-IL4Rα group compared to control group across various outcomes, including AER, FEV1, ACQ, AQLQ, FENO, AEs, and SAEs. In terms of efficacy, anti-IL4Rα group demonstrated a significant reduction in AER (RR: 0.51, 95% CI: 0.42–0.62, *P* < 0.0001, *I*^2^ = 87.6%) (Supplementary Figure S5A). In the lung function test, FEV1 values showed a significant improvement in the anti-IL4Rα group (MD: 0.15, 95% CI: 0.15–0.15, *P* < 0.0001, *I*^2^ = 0%) (Supplementary Figure S6A). Furthermore, regarding the PROs, the ACQ scores decreased significantly in the anti-IL4Rα group (MD: −0.33, 95% CI: −0.34 to −0.31, *P* < 0.0001, *I*^2^ = 0%) (Supplementary Figure S6B), and the AQLQ scores increased significantly (MD: 0.27, 95% CI: 0.21–0.34, *P* < 0.0001, *I*^2^ = 57%) (Supplementary Figure S6C). Additionally, the FENO levels significantly decreased in the anti-IL4Rα group (MD: −32.02, 95% CI: −35.40 to −28.63, *P* < 0.0001, *I*^2^ = 0%) (Supplementary Figure S6D). In terms of safety, the analysis revealed no significant differences in the risk of AEs and SAEs between the two groups (RR: 1.00, 95% CI: 0.98–1.02, *P* = 0.9402, *I*^2^ = 0%; RR: 1.10, 95% CI: 0.90–1.36, *P* = 0.3418, *I*^2^ = 0%) (Supplementary Figures S5B,C).

There was high heterogeneity in AER and AQLQ scores, which indicated that these two outcomes vary significantly across the included studies. However, as demonstrated in the forest plots, each study of every outcome was located on the same side of the axis or intersected with the line of no effect, which exhibited a potential consistent trend.

#### 3.5.4 Anti-TSLP treatment

The meta-analysis assessed the efficacy and safety of anti-TSLP group compared with control group across various outcomes, including AER, FEV1, ACQ, AQLQ, FENO, blood eosinophils, AEs, and SAEs. In terms of efficacy, anti-TSLP group resulted in a significant decrease in AER compared to the control group (RR: 0.47, 95% CI: 0.40–0.55, *P* < 0.0001, *I*^2^ = 0%) (Supplementary Figure S7A). Regarding the lung function, the anti-TSLP group demonstrated a significant improvement on FEV1 (MD: 0.16, 95% CI: 0.13–0.19, *P* < 0.0001, *I*^2^ = 0%) (Supplementary Figure S8A). Moreover, analysis of PROs showed that the ACQ scores decreased significantly in the anti-TSLP group (MD: −0.33, 95% CI: −0.34 to −0.32, *P* < 0.0001, *I*^2^ = 0%) (Supplementary Figure S8B), and the AQLQ scores increased significantly (MD: 0.34, 95% CI: 0.33–0.35, *P* < 0.0001, *I*^2^ = 0%) (Supplementary Figure S8C). Furthermore, the FENO levels and blood eosinophils showed a significant decline in the anti-TSLP group (MD: −12.13, 95% CI: −13.31 to −10.94, *P* < 0.0001, *I*^2^ = 34.7%; MD: −138.92, 95% CI: −149.18 to −128.66, *P* < 0.0001, *I*^2^ = 0%) (Supplementary Figures S8D,E). In terms of safety, no significant difference was observed in the risk of AEs between the two groups (RR: 0.81, 95% CI: 0.63–1.05, *P* = 0.0129, *I*^2^ = 66.8%) (Supplementary Figure S7B). However, the incidence of SAEs in the anti-TSLP group was significantly lower than that in the control group (RR: 0.78, 95% CI: 0.61–0.98, *P* = 0.0358, *I*^2^ = 71.1%) (Supplementary Figure S7C).

High heterogeneity was observed in AEs and SAEs. As shown in the forest plots, the trends of the included studies were inconsistent. The heterogeneity of other outcomes was relatively low, and each study for every outcome was located on the same side of the axis.

### 3.6 Sensitivity analysis

In order to evaluate the influence of each study on overall outcomes, we conducted the sensitivity analysis. In the overall evaluation of biologics, high heterogeneity was detected in AER, FEV1, AQLQ scores, FENO levels, AEs, and SAEs. Sensitivity analysis found that the absence of each study cannot significantly change the overall values, and it verified the stability of results in this umbrella review (Supplementary Figures S9–S14). In the evaluation of anti-IgE treatment, high heterogeneity was present in SAEs. Sensitivity analysis demonstrated that the absence of SAEs reported by the first three authors significantly altered the overall values (Supplementary Figure S15). In the evaluation of anti-IL5/5Rα treatment, high heterogeneity was observed on ACQ scores and AEs. Sensitivity analysis showed the stability of ACQ scores among different SR/MAs. However, the absence of the three groups in the study of Agache et al. (26) would significantly change the overall values of AEs (Supplementary Figures S16, S17). In the evaluation of anti-IL4Rα treatment, high heterogeneity was detected in AER and AQLQ scores. Sensitivity analyses found that omitting any single study did not significantly change the overall values (Supplementary Figures S18, S19). In the evaluation of anti-TSLP treatment, there was high heterogeneity on AEs and SAEs, and sensitivity analysis found that the absence of some studies would significantly affect the overall values (Supplementary Figures S20, S21).

## 4 Discussion

Despite the fact that previous SR/MAs have already proven the favorable efficacy and safety of biologics for severe asthma (15, 16, 19), some study results were inconsistent among different SR/MAs. Meanwhile, the methodological quality of these SR/MAs was unclear, which may have an impact on clinical decisions. Umbrella reviews can assess the quality of SR/MAs and systematically synthesize their findings. As far as we know, this is the first umbrella review to evaluate the methodological quality of relevant SR/MAs and reassess the efficacy and safety of biologics for severe asthma.

In general, patients who received biologic therapy had fewer AER and asthma-related hospitalizations, better FEV1, lower ACQ scores, higher AQLQ scores, lower FENO levels than control group. Moreover, overall, biologics showed an acceptable safety profile. Sensitivity analyses were conducted for all outcomes with high heterogeneity. It illustrated that omitting any single study cannot significantly change the overall values, thus verifying the stability of the results in this umbrella review.

Omazumab is a recombinant humanized anti-IgE monoclonal antibody with a dual inhibitory effect. On the one hand, it can target and bind free IgE, thereby reducing IgE binding to high-affinity IgE receptors on basophils and mast cells. On the other hand, it also inhibits the expression of IgE receptors on mast cells (35, 36). Analyses demonstrated that anti-IgE treatment had clinically significant effects on AER, asthma-related hospitalizations, FEV1 changes, ACQ and AQLQ scores. These outcomes showed relatively low heterogeneity, indicating the potential consistency of these results across different SR/MAs. Regarding safety, there was no significant difference in AEs between the two groups, and fewer SAEs occurred in the anti-IgE group. We observed high heterogeneity in SAEs. Sensitivity analysis demonstrated that the absence of SAEs reported by the first three authors significantly altered the overall values. The possible explanation is that the populations in these three studies were relatively younger. Although current research does not support the notion that the incidence of SAEs in young people treated with biologics for asthma is definitively lower, young individuals may possess potential advantages that could reduce the occurrence of SAEs. Firstly, young individuals exhibit greater efficiency in modulating immune responses, thereby reducing the serious problems caused by immune-related AEs (37). Secondly, young people tend to have better compliance with asthma treatment and are more adept at using modern technology for self-management, which can contribute to reducing the incidence of SAEs (38).

IL-5 is a key cytokine in the eosinophilic inflammatory pathway. Previous studies have shown that IL-5 plays a key role in the survival, recruitment and stimulation of eosinophils (39). Anti-il-5/IL-5R monoclonal antibodies can inhibit the increase of eosinophils by targeting IL-5 or the alpha subunit of the IL-5 receptor (40). Our study showed that anti-IL5/5Rα treatment was associated with better clinical effects. In terms of safety, patients treated with anti-IL5/5Rα treatment reported similar AEs and fewer SAEs compared to placebo. High heterogeneity was observed on ACQ scores and AEs. Sensitivity analysis demonstrated the stability of ACQ scores among different SR/MAs. However, sensitivity analysis also showed that the absence of the three groups in the study of Agache et al. (26) would significantly change the overall values of AEs. As illustrated in the forest plot, the three groups all tended toward the right side of the axis. In the study of Agache et al., it was mentioned that the possible reason is that they limited their assessment to drug-related AEs, excluded asthma worsening events, and assessed solely as efficacy measures (26).

Dupilumab is a monoclonal antibody targeting the alpha subunit of the IL-4 receptor. By inhibiting downstream signaling, it can reduce eosinophilic recruitment and activation, thereby ameliorating airway hyperresponsiveness and airway remodeling (36, 41). On the basis of the results of this umbrella review, anti-IL4Rα treatment significantly improved each efficacy outcome, and the risks of AEs and SAEs were similar to the control group. High heterogeneity was observed in AER and AQLQ scores. Sensitivity analyses found that omitting any single study did not significantly change the overall values, thus proving the stability of these two outcomes. Nevertheless, four of the seven included studies had low methodological quality. Thus, more high-quality systematic reviews are needed to further verify the effects of anti-IL4Rα treatment on patients with severe asthma.

TSLP, which targets immune cells to release pro-inflammatory cytokines, plays an important role in the pathogenesis of asthma. Tezepelumab is a humanized monoclonal antibody against TSLP to prevent asthma exacerbations and improve asthma control. Because it acts upstream in the inflammatory cascade, tezepelumab may be suitable for a broad range of patients with severe uncontrolled asthma, regardless of phenotype or T2 biomarker status (42, 43). Analysis showed that patients treated with anti-TSLP treatment received better clinical effects than the control group. Regarding safety, patients had similar risk of AEs and lower risk of SAEs than those treated with placebo. There was high heterogeneity on AEs and SAEs, and sensitivity analysis showed that the absence of some studies would significantly affect the overall values. As only four SR/MAs of anti-TSLP treatment were included in this umbrella review, sufficient data were not available for subgroup analysis. In addition, the methodological quality of the four systematic reviews was low or critically low, and the interpretation of these research evidence needs to be cautious.

Given the paucity of SR/MAs focusing on pediatric populations, this umbrella review focused on adult populations. However, it is noteworthy that several biologics have demonstrated efficacy in severe pediatric asthma. In previous SR/MAs, omalizumab reduces asthma exacerbations, the dose of inhaled corticosteroids, and the incidence of serious adverse events in children and adolescents with severe asthma (21, 44). Furthermore, according to the 2025 Global Initiative for Asthma, omalizumab is recommended for patients aged ≥ 6 years with severe allergic asthma, mepolizumab for patients aged ≥ 6 years with severe eosinophilic asthma, benralizumab for patients aged ≥ 12 years with severe eosinophilic asthma, dupilumab for patients aged ≥ 6 years with severe eosinophilic/Type 2 asthma, and tezepelumab for patients aged ≥ 12 years with severe asthma (45).

However, this study has some limitations that should be acknowledged. Firstly, we only focused on meta-analyses that included RCTs, which may have omitted important data. Secondly, a total of 17 studies had low or critically low methodological quality that could have affected our results. The low quality was mainly due to the lack of a list of excluded literature and reasons for exclusion. Finally, AEs and SAEs in the anti-TSLP treatment showed high heterogeneity. However, only four SR/MAs are available, and there is insufficient data to conduct subgroup analyses to explore the sources of heterogeneity. Further in-depth research on the safety of anti-TSLP treatment will be required in the future.

## 5 Conclusion

This umbrella review indicated that anti-IgE treatment, anti-IL5/5Rα treatment, anti-IL4Rα treatment, and anti-TSLP treatment may be beneficial to patients with severe asthma. However, further high-quality primary studies and SR/MAs are still needed to further demonstrate these potential benefits, because the SR/MAs included in this study were mainly low or critically low methodological quality.
