# Supplementary material for: The efficacy and safety of biologics for patients with severe asthma: an umbrella review of systematic reviews and meta-analyses
Source: Front Med (Lausanne). 2025 May 30;12:1573596. doi: 10.3389/fmed.2025.1573596 (PMC12162592; doi:10.3389/fmed.2025.1573596)
Supplement: Supplementary file 1 [file Data_Sheet_1.docx]

Supplementary Material

# Supplementary Figures and Tables

## Supplementary Figures

##
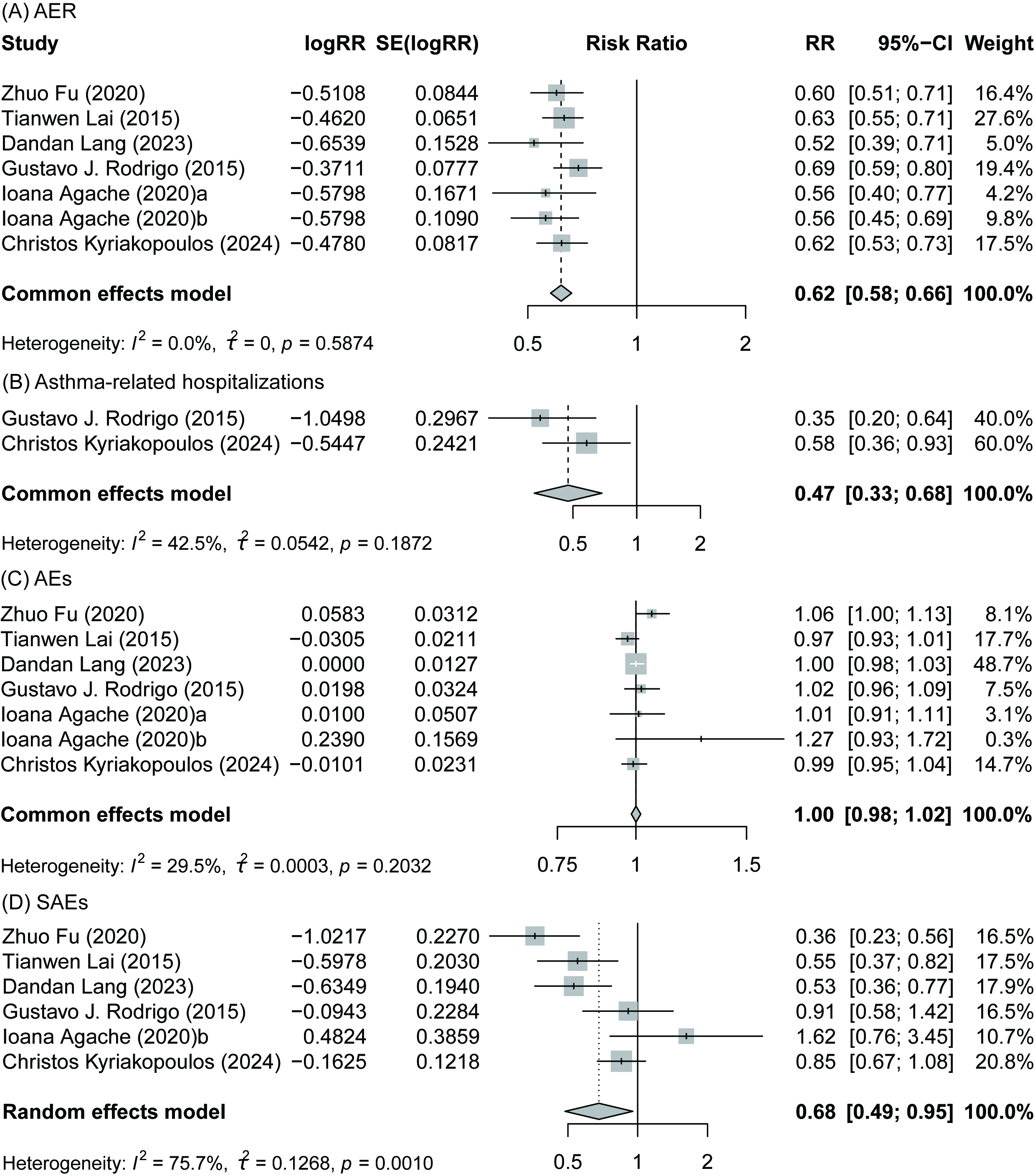


**Supplementary Figure S1.** Forest plot of risk ratios for discrete variables comparing anti-immunoglobulin E treatment and control treatment. **(A)** Forest plot of asthma exacerbation rate comparing anti-immunoglobulin E treatment and control treatment. **(B)** Forest plot of asthma-related hospitalizations comparing anti-immunoglobulin E treatment and control treatment. **(C)** Forest plot of adverse events comparing anti-immunoglobulin E treatment and control treatment. **(D)** Forest plot of serious adverse events comparing anti-immunoglobulin E treatment and control treatment.


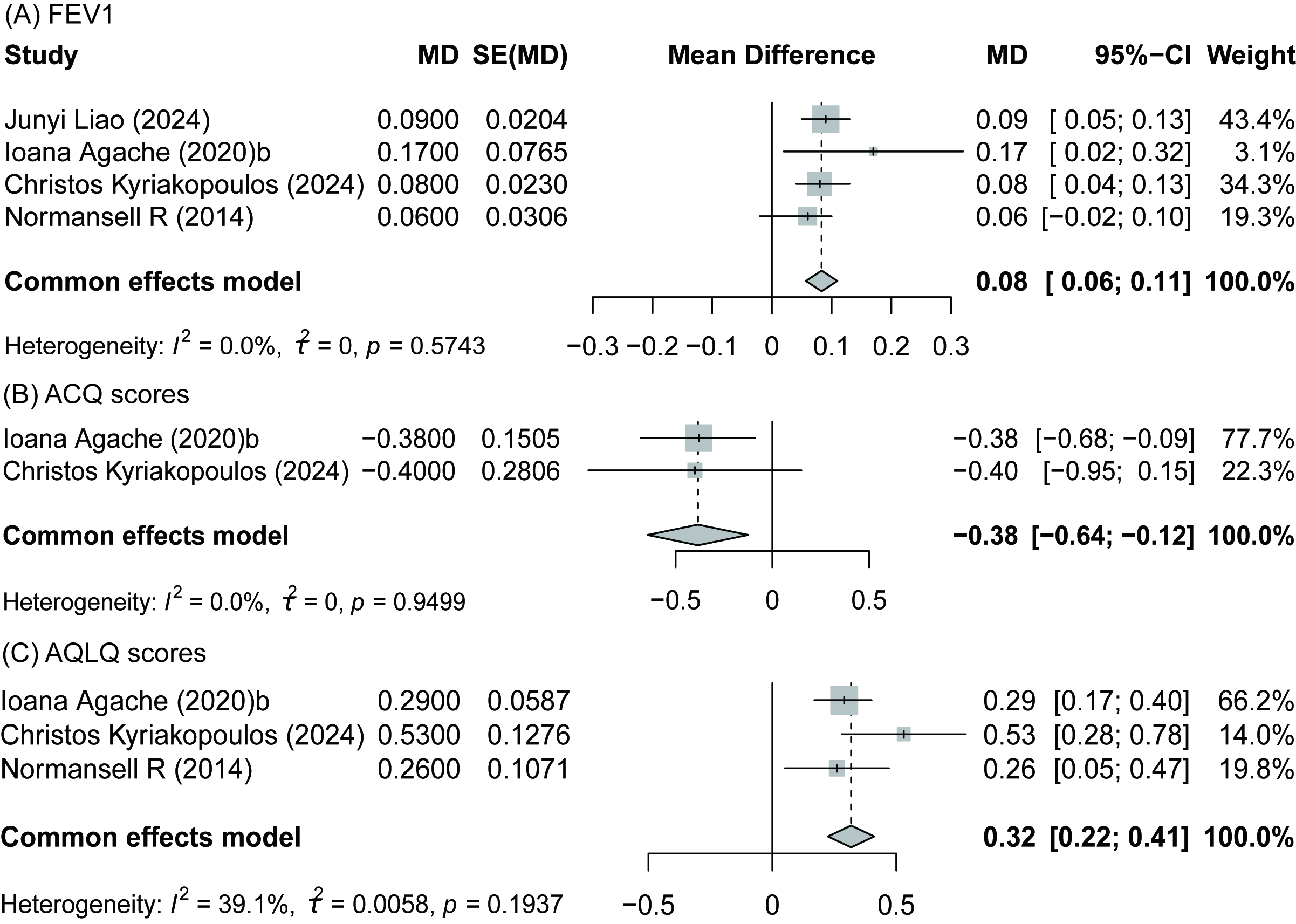


**Supplementary Figure S2.** Forest plot of mean differences for continuous variables comparing anti-immunoglobulin E treatment and control treatment. **(A)** Forest plot of forced expiratory volume in 1 s comparing anti-immunoglobulin E treatment and control treatment. **(B)** Forest plot of asthma control questionnaire scores comparing anti-immunoglobulin E treatment and control treatment. **(C)** Forest plot of asthma quality of life questionnaire scores comparing anti-immunoglobulin E treatment and control treatment.


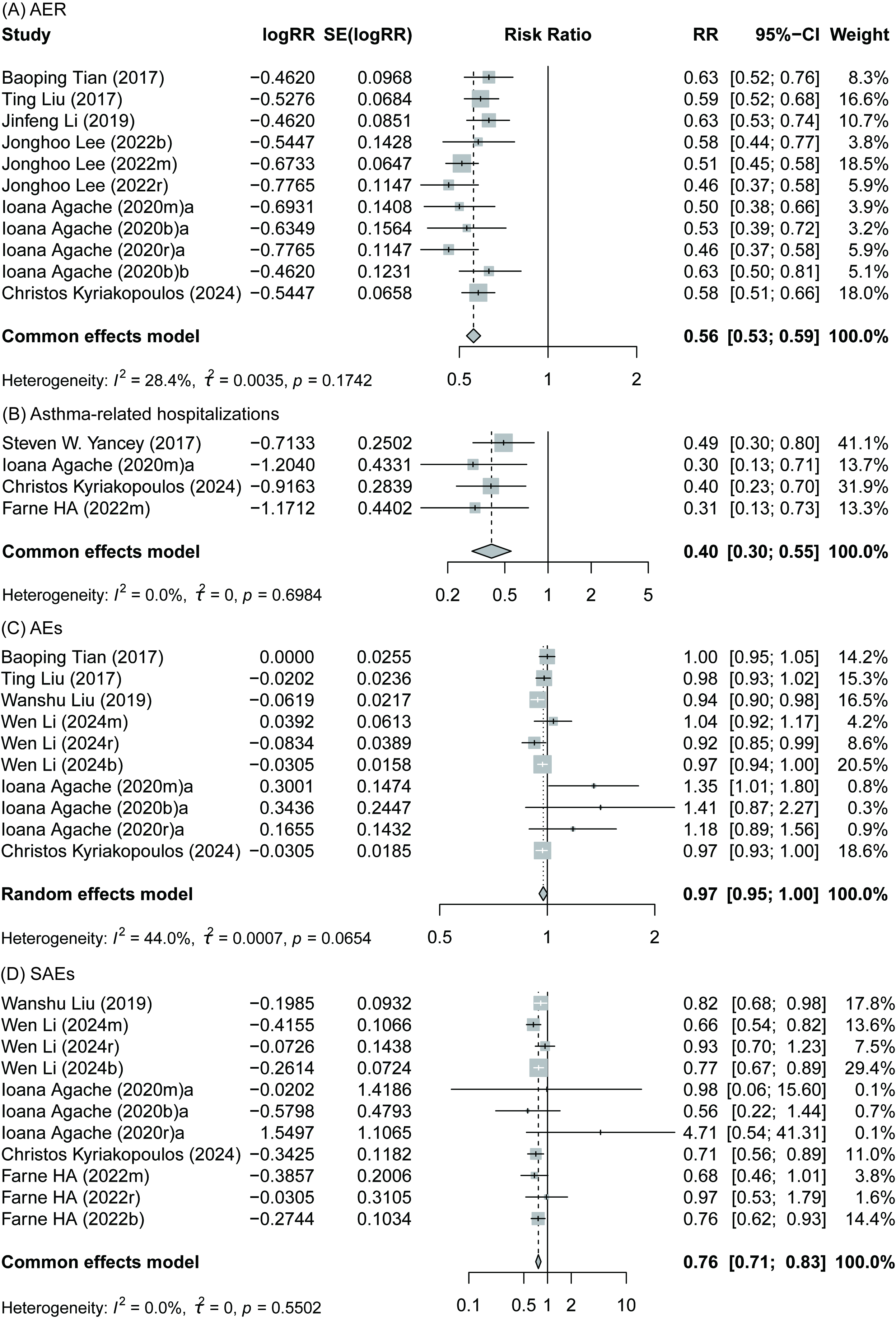


**Supplementary Figure S3.** Forest plot of risk ratios for discrete variables comparing anti-interleukin-5/5Rα treatment and control treatment. **(A)** Forest plot of asthma exacerbation rate comparing anti-interleukin-5/5Rα treatment and control treatment. **(B)** Forest plot of asthma-related hospitalizations comparing anti-interleukin-5/5Rα treatment and control treatment. **(C)** Forest plot of adverse events comparing anti-interleukin-5/5Rα treatment and control treatment. **(D)** Forest plot of serious adverse events comparing anti-interleukin-5/5Rα treatment and control treatment. Abbreviations: m: mepolizumab, b: benralizumab, r: reslizumab.


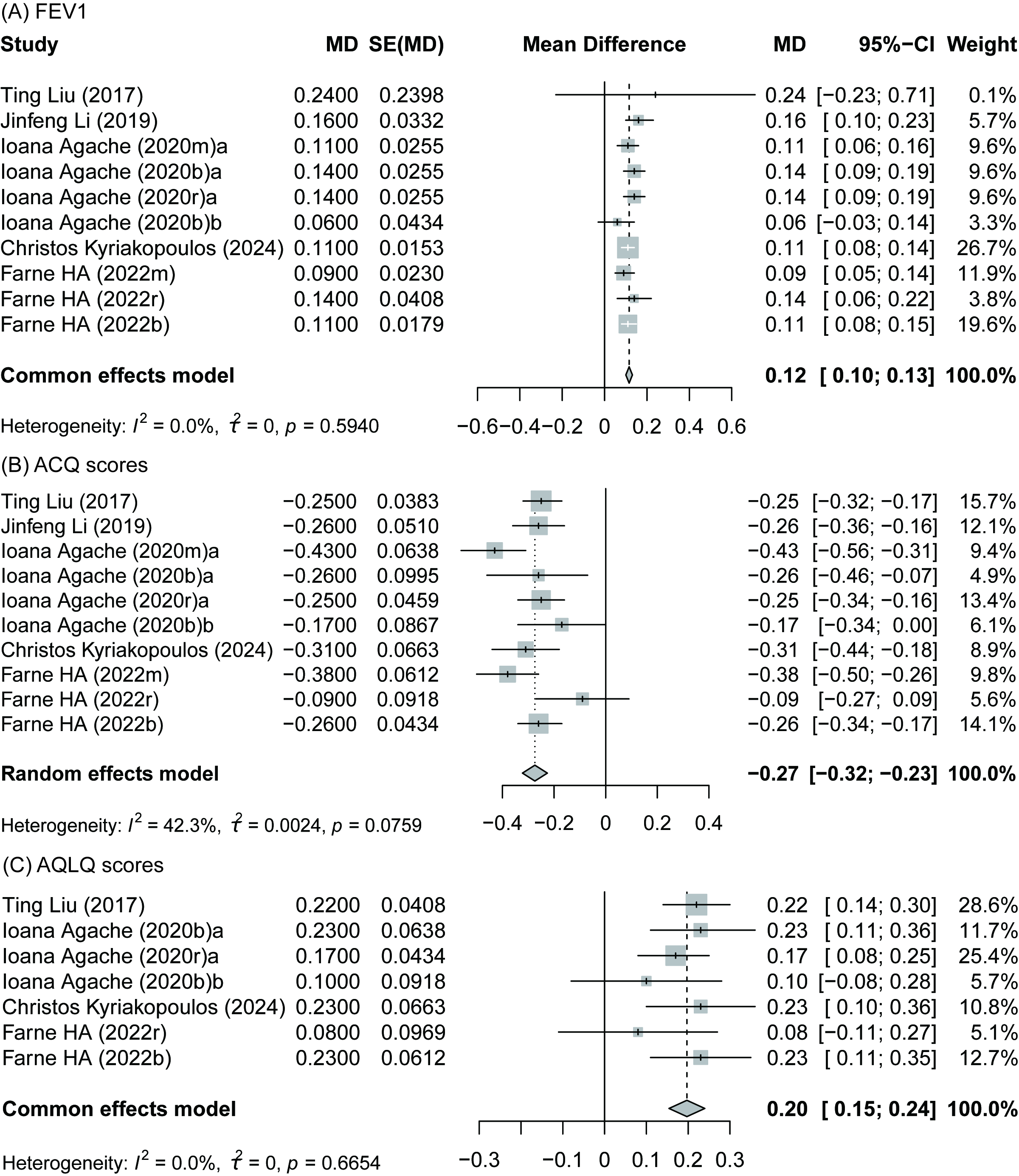


**Supplementary Figure S4.** Forest plot of mean differences for continuous variables comparing anti-interleukin-5/5Rα treatment and control treatment. **(A)** Forest plot of forced expiratory volume in 1 s comparing anti-interleukin-5/5Rα treatment and control treatment. **(B)** Forest plot of asthma control questionnaire scores comparing anti-interleukin-5/5Rα treatment and control treatment. **(C)** Forest plot of asthma quality of life questionnaire scores comparing anti-interleukin-5/5Rα treatment and control treatment. Abbreviations: m: mepolizumab, b: benralizumab, r: reslizumab.


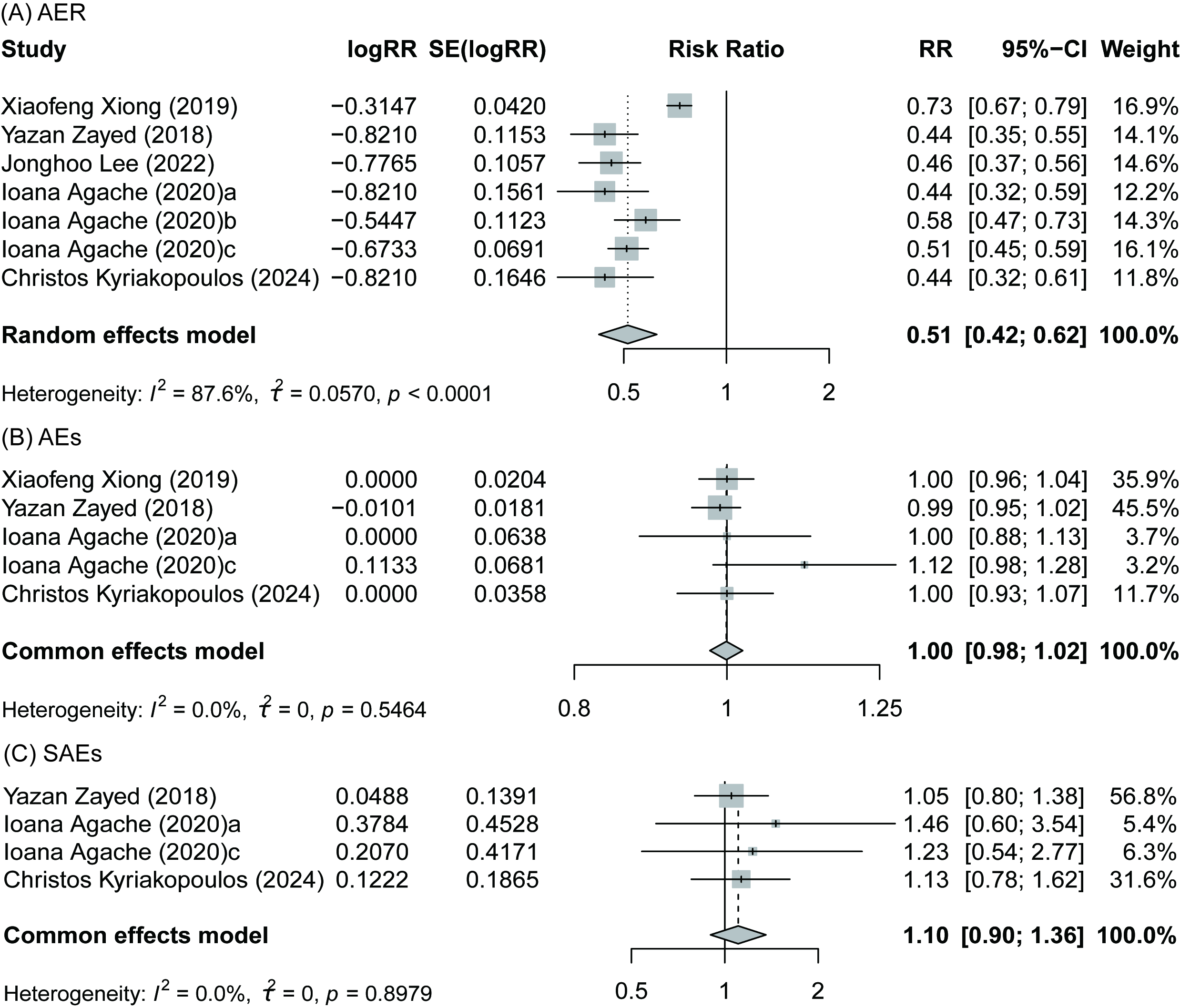


**Supplementary Figure S5.** Forest plot of risk ratios for discrete variables comparing anti-interleukin-4Rα treatment and control treatment. **(A)** Forest plot of asthma exacerbation rate comparing anti-interleukin-4Rα treatment and control treatment. **(B)** Forest plot of adverse events comparing anti-interleukin-4Rα treatment and control treatment. **(C)** Forest plot of serious adverse events comparing anti-interleukin-4Rα treatment and control treatment.


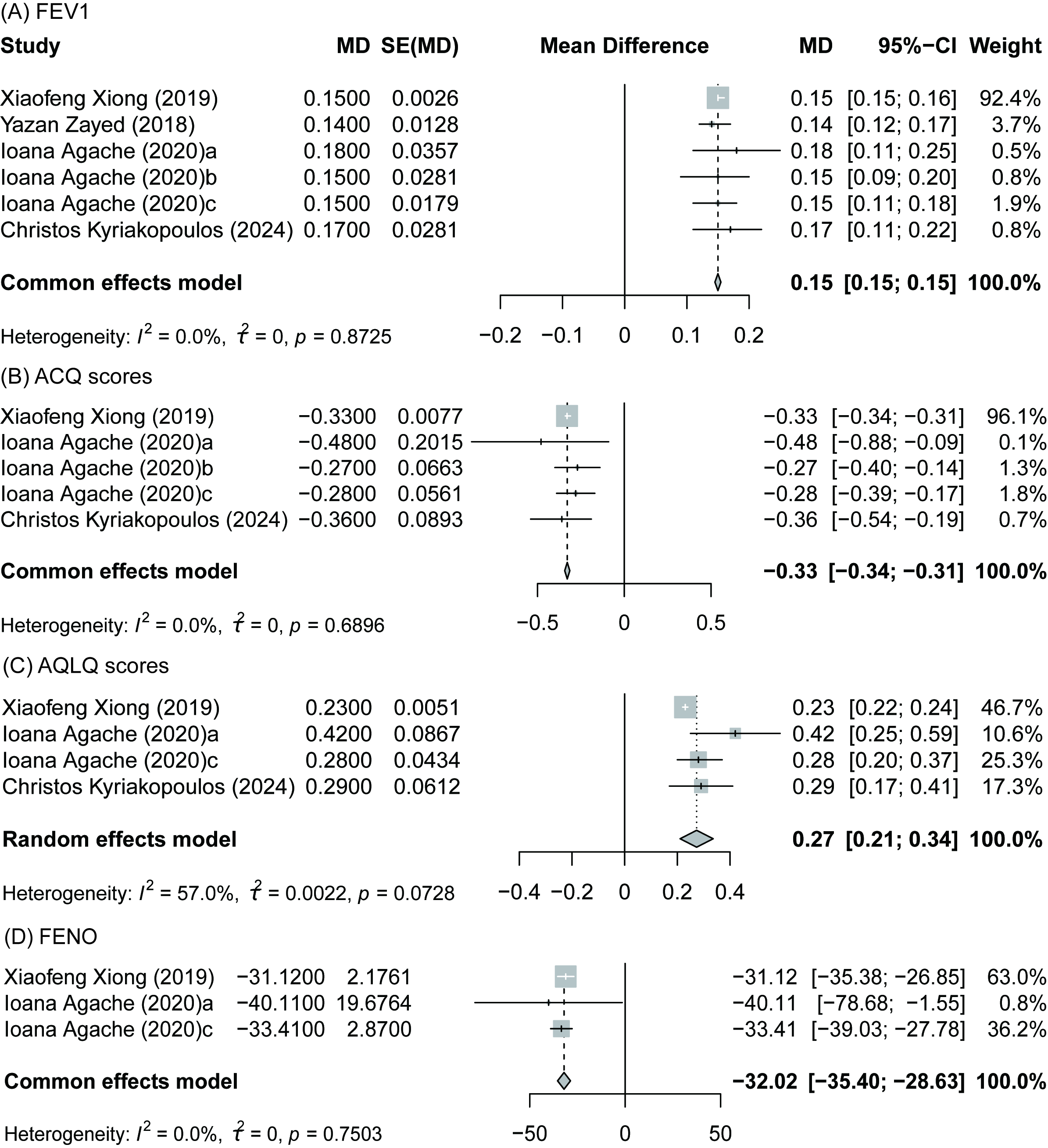


## **Supplementary Figure S6.** Forest plot of mean differences for continuous variables comparing anti-interleukin-4Rα treatment and control treatment. **(A)** Forest plot of forced expiratory volume in 1 s comparing anti-interleukin-4Rα treatment and control treatment. **(B)** Forest plot of asthma control questionnaire scores comparing anti-interleukin-4Rα treatment and control treatment. **(C)** Forest plot of asthma quality of life questionnaire scores comparing anti-interleukin-4Rα treatment and control treatment. **(D)** Forest plot of fractional exhaled nitric oxide comparing anti-interleukin-4Rα treatment and control treatment.


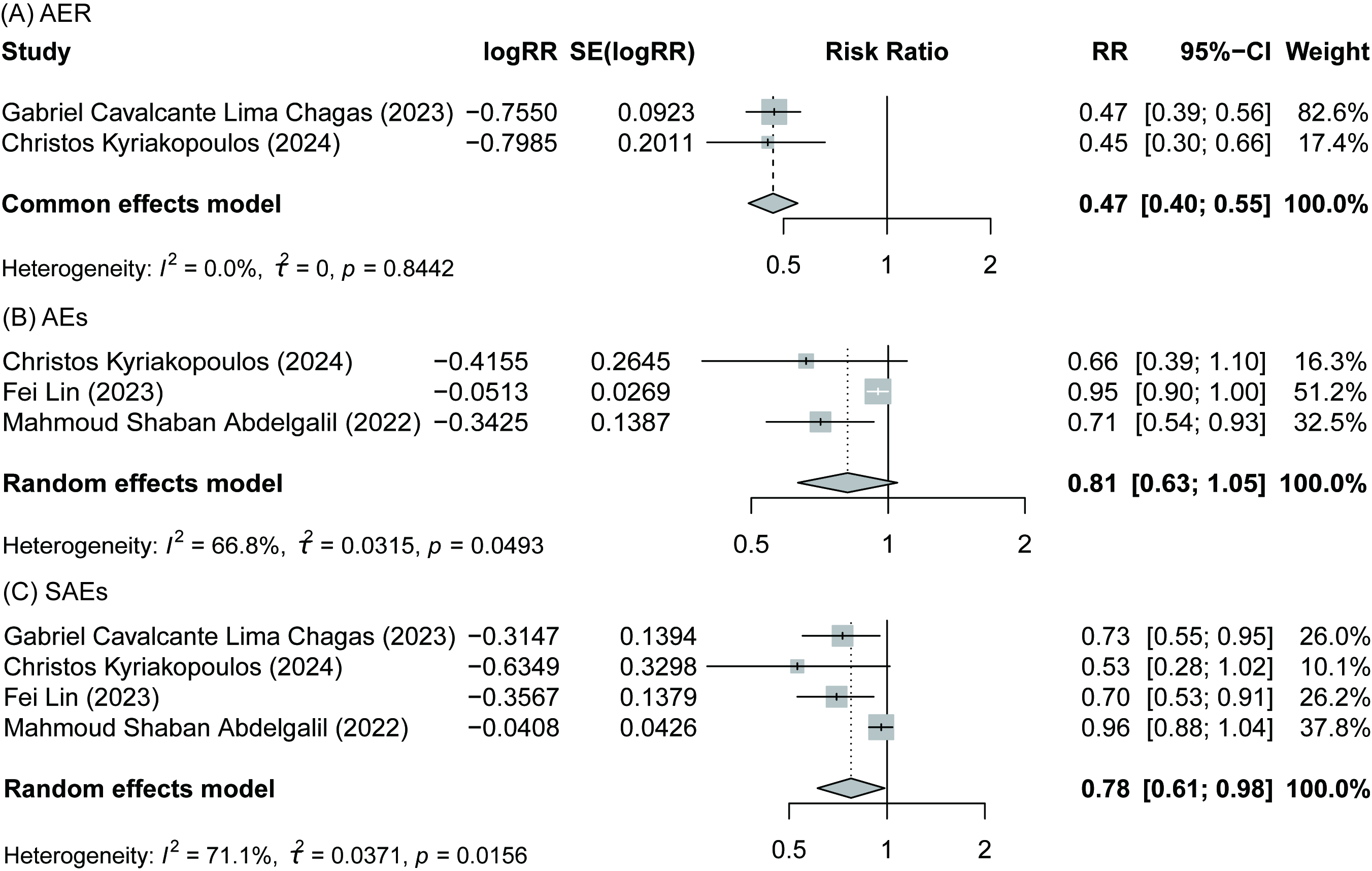


**Supplementary Figure S7.** Forest plot of risk ratios for discrete variables comparing anti-thymic stromal lymphopoietin treatment and control treatment. **(A)** Forest plot of asthma exacerbation rate comparing anti-thymic stromal lymphopoietin treatment and control treatment. **(B)** Forest plot of adverse events comparing anti-thymic stromal lymphopoietin treatment and control treatment. **(C)** Forest plot of serious adverse events comparing anti-thymic stromal lymphopoietin treatment and control treatment.


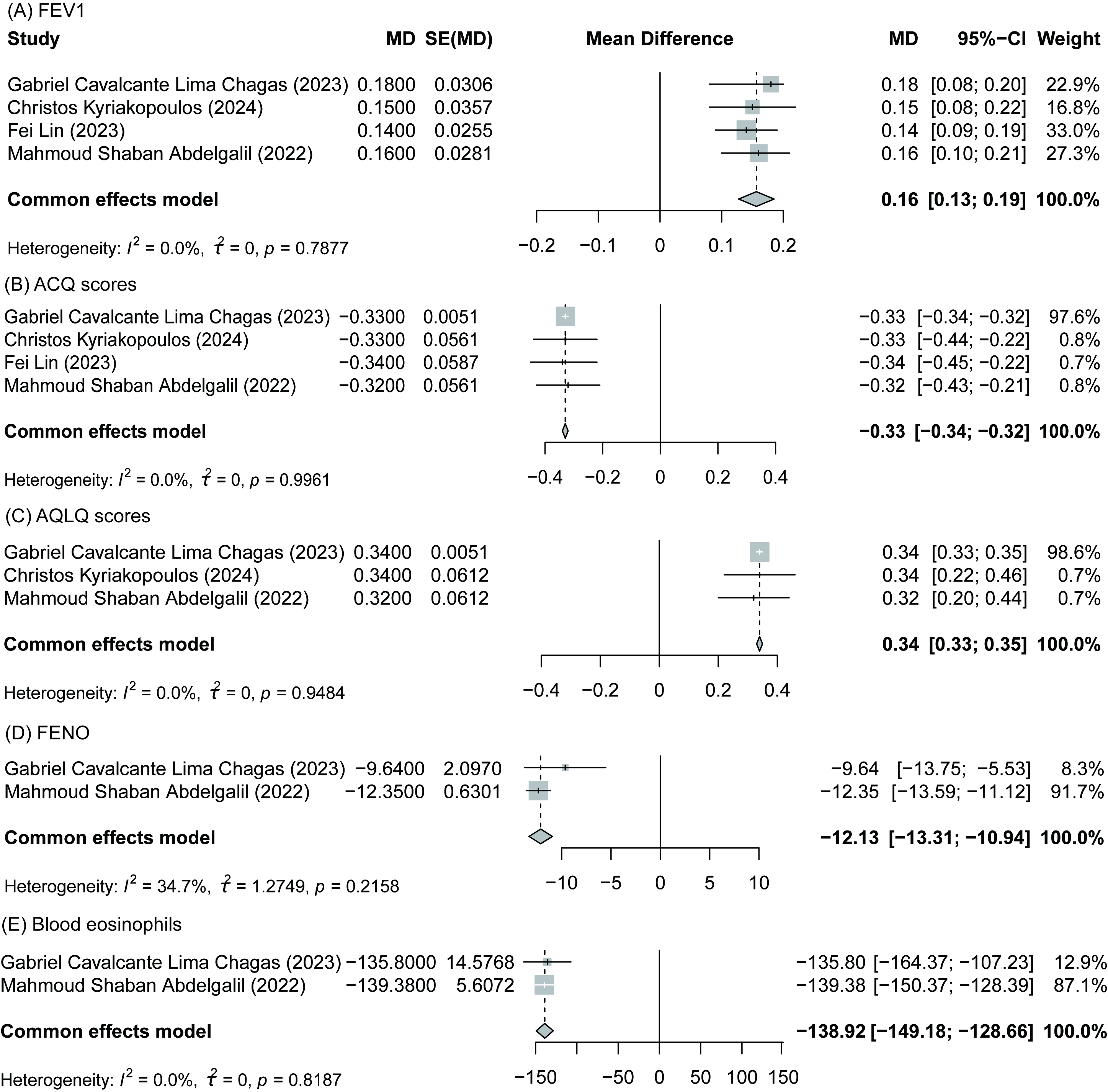


**Supplementary Figure S8.** Forest plot of mean differences for continuous variables comparing anti-thymic stromal lymphopoietin treatment and control treatment. **(A)** Forest plot of forced expiratory volume in 1 s comparing anti-thymic stromal lymphopoietin treatment and control treatment. **(B)** Forest plot of asthma control questionnaire scores comparing anti-thymic stromal lymphopoietin treatment and control treatment. **(C)** Forest plot of asthma quality of life questionnaire scores comparing anti-thymic stromal lymphopoietin treatment and control treatment. **(D)** Forest plot of fractional exhaled nitric oxide comparing anti-thymic stromal lymphopoietin treatment and control treatment. **(E)** Forest plot of blood eosinophils comparing anti-thymic stromal lymphopoietin treatment and control treatment.


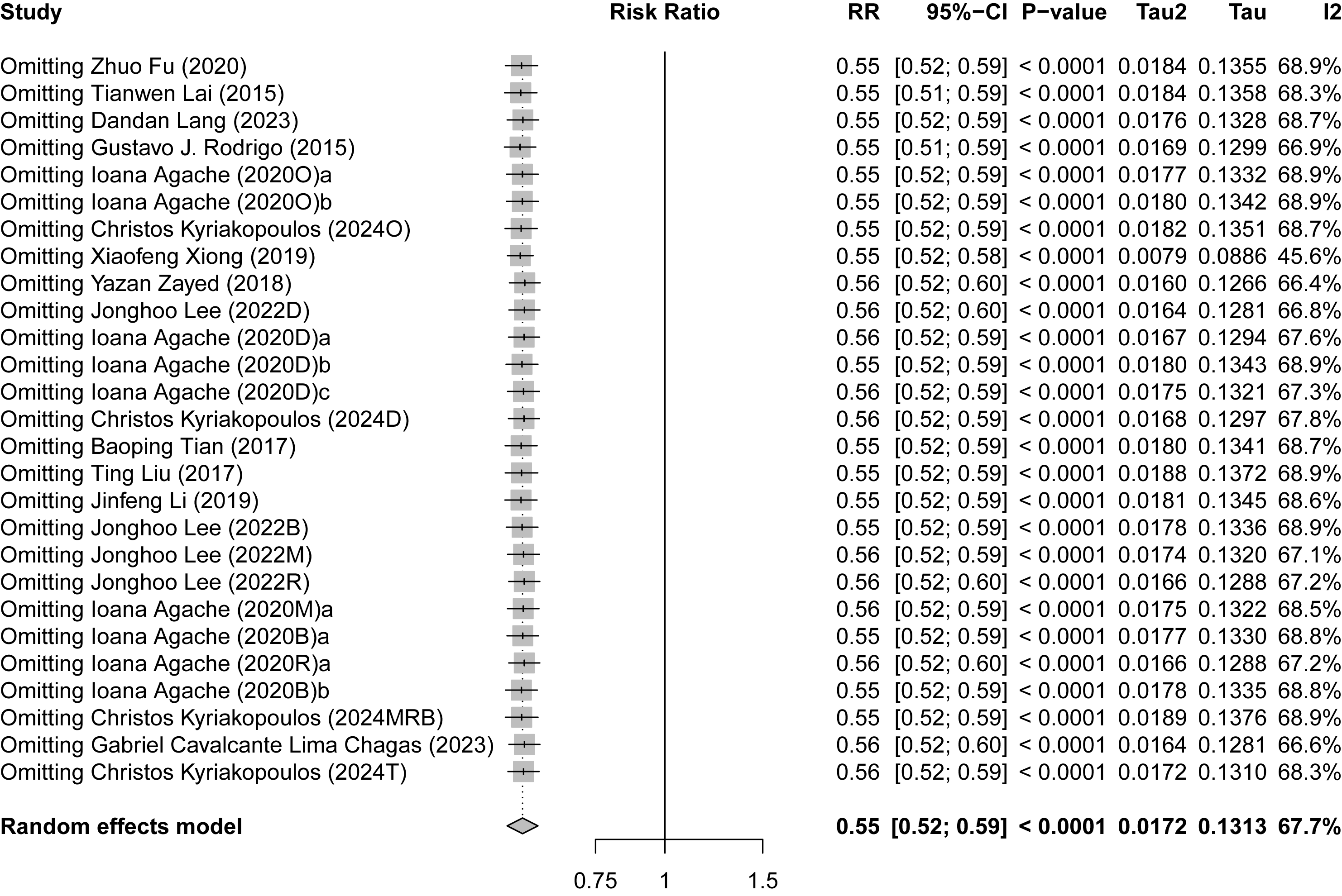


**Supplementary Figure S9.** Sensitivity analysis of risk ratios of asthma exacerbation rate in severe asthma treated with biologics. Abbreviations: O: omalizumab, D: dupilumab, M: mepolizumab, R: reslizumab, B: benralizumab, T: tezepelumab.


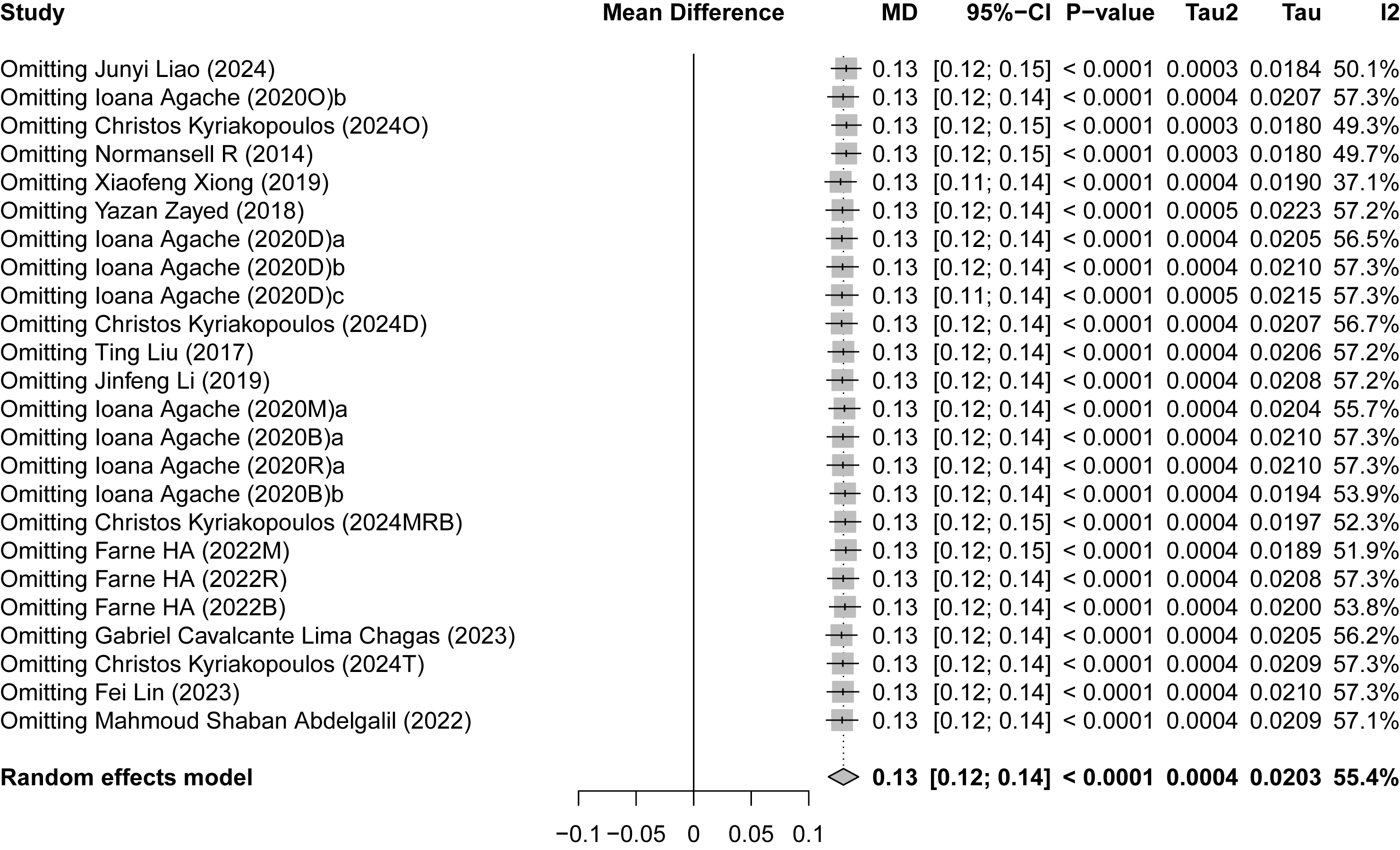


**Supplementary Figure S10.** Sensitivity analysis of mean differences of forced expiratory volume in 1 s in severe asthma treated with biologics. Abbreviations: O: omalizumab, D: dupilumab, M: mepolizumab, R: reslizumab, B: benralizumab, T: tezepelumab.


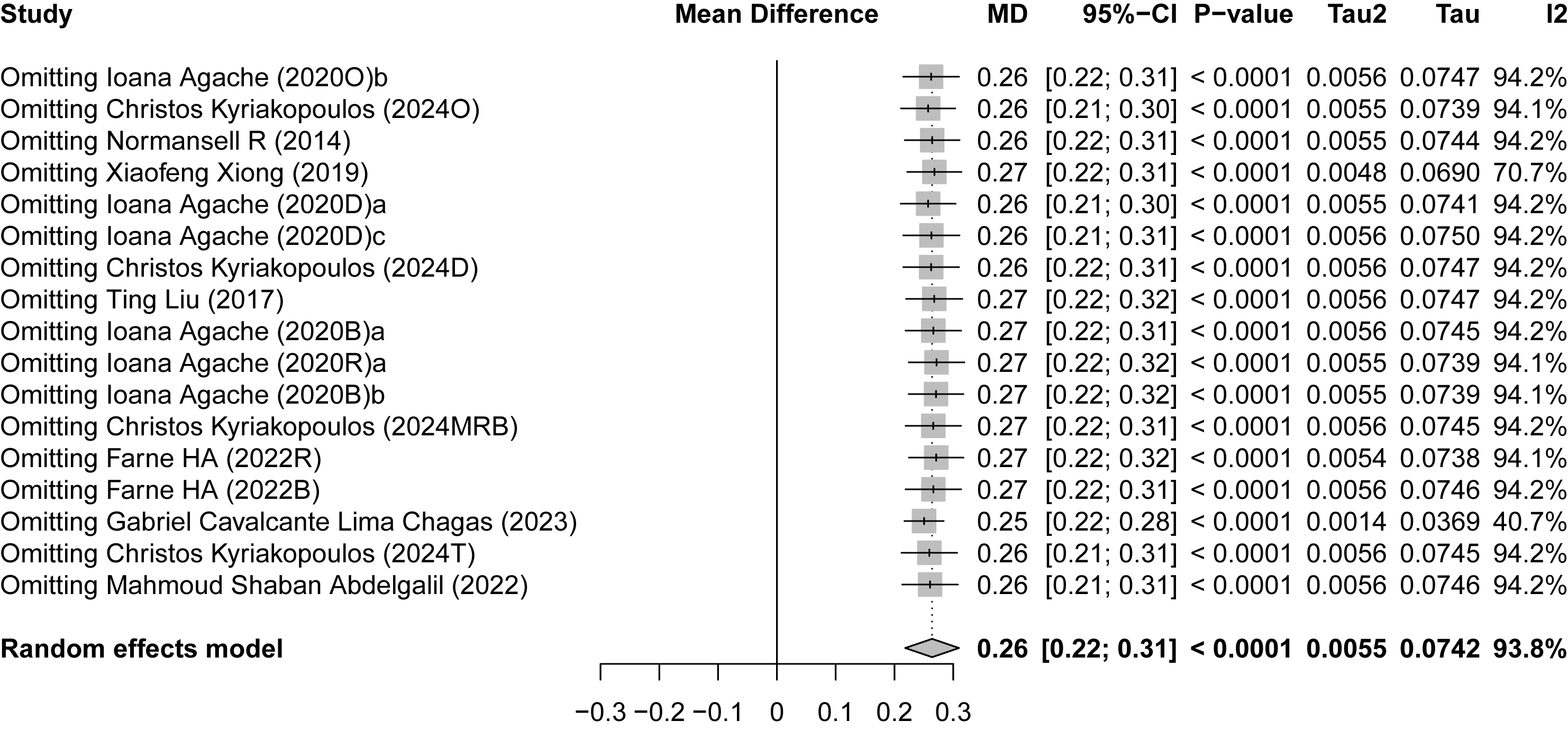


**Supplementary Figure S11.** Sensitivity analysis of mean differences of asthma quality of life questionnaire scores in severe asthma treated with biologics. Abbreviations: O: omalizumab, D: dupilumab, M: mepolizumab, R: reslizumab, B: benralizumab, T: tezepelumab.


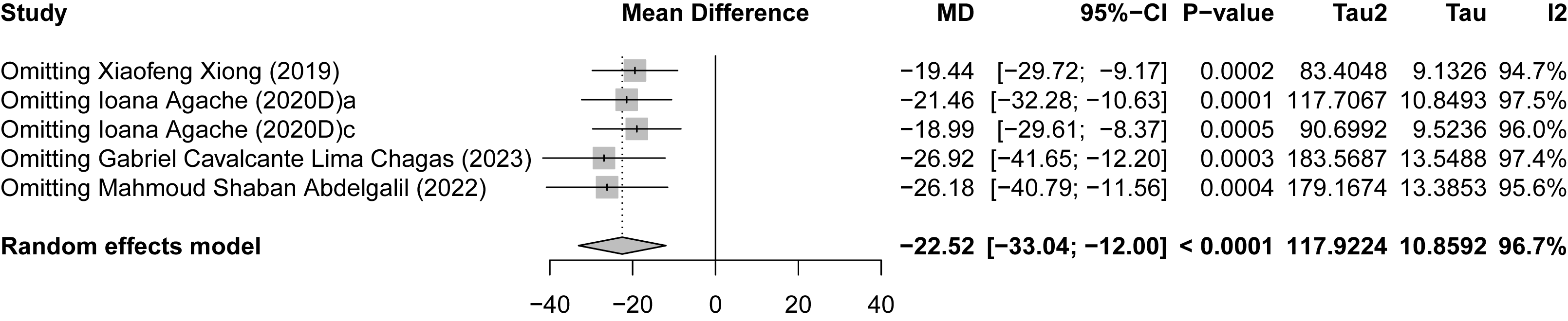


**Supplementary Figure S12.** Sensitivity analysis of mean differences of fractional exhaled nitric oxide in severe asthma treated with biologics. Abbreviations: D: dupilumab.


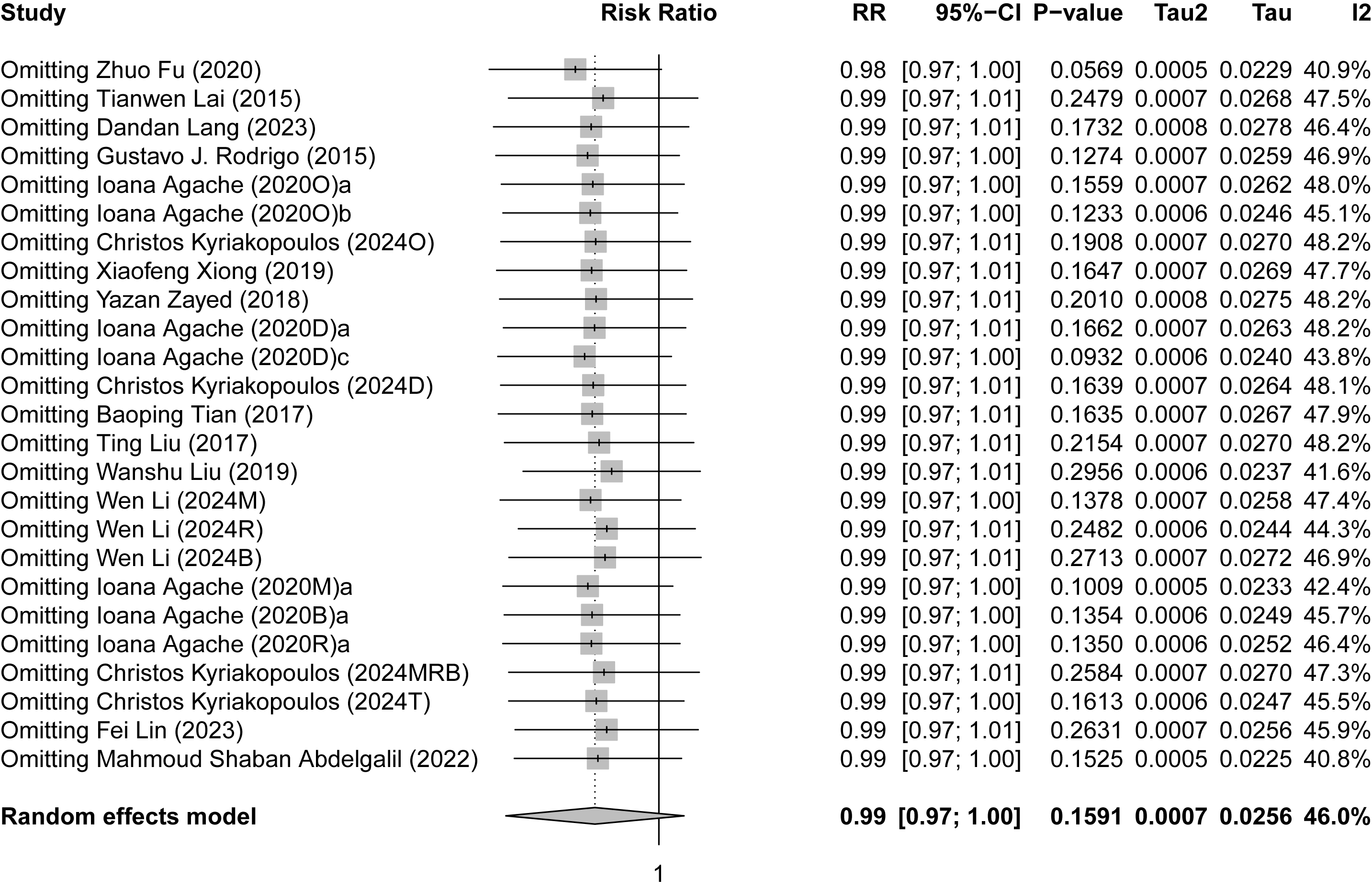


**Supplementary Figure S13.** Sensitivity analysis of risk ratios of adverse events in severe asthma treated with biologics. Abbreviations: O: omalizumab, D: dupilumab, M: mepolizumab, R: reslizumab, B: benralizumab, T: tezepelumab.


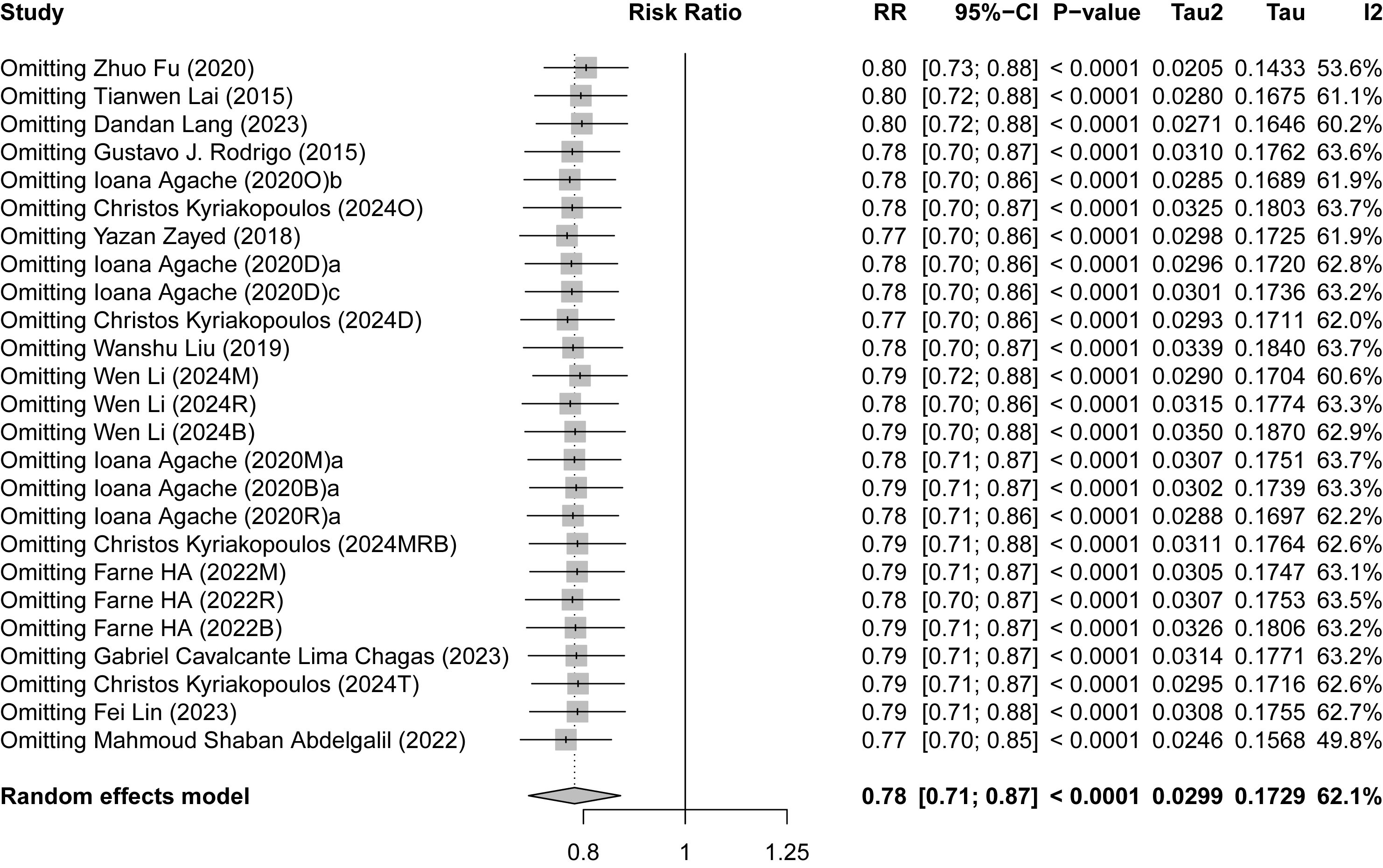


**Supplementary Figure S14.** Sensitivity analysis of risk ratios of serious adverse events in severe asthma treated with biologics. Abbreviations: O: omalizumab, D: dupilumab, M: mepolizumab, R: reslizumab, B: benralizumab, T: tezepelumab.


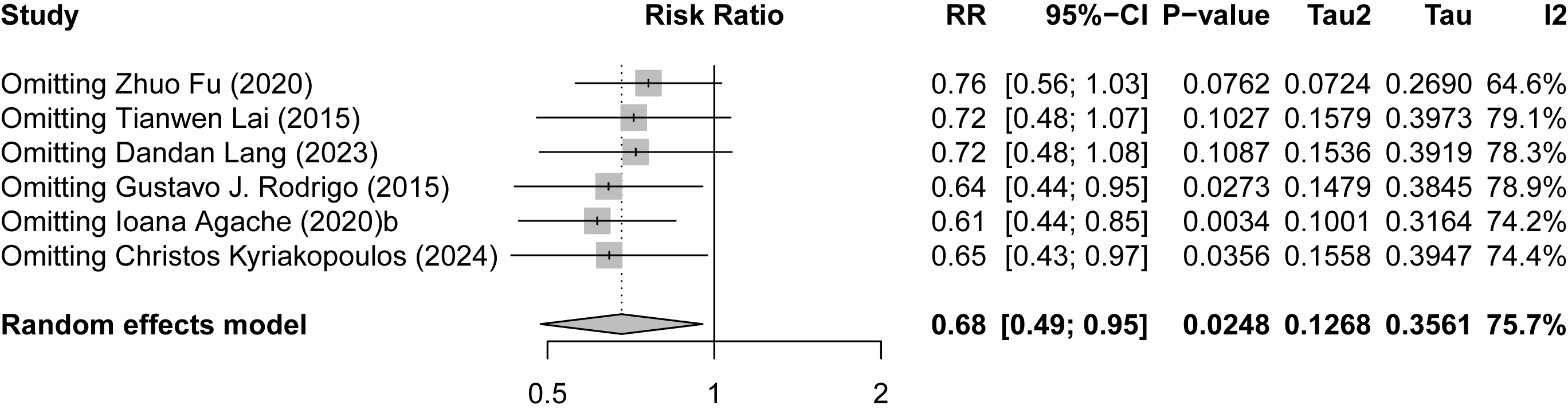


**Supplementary Figure S15.** Sensitivity analysis of risk ratios of serious adverse events in severe asthma treated with anti-immunoglobulin E treatment.


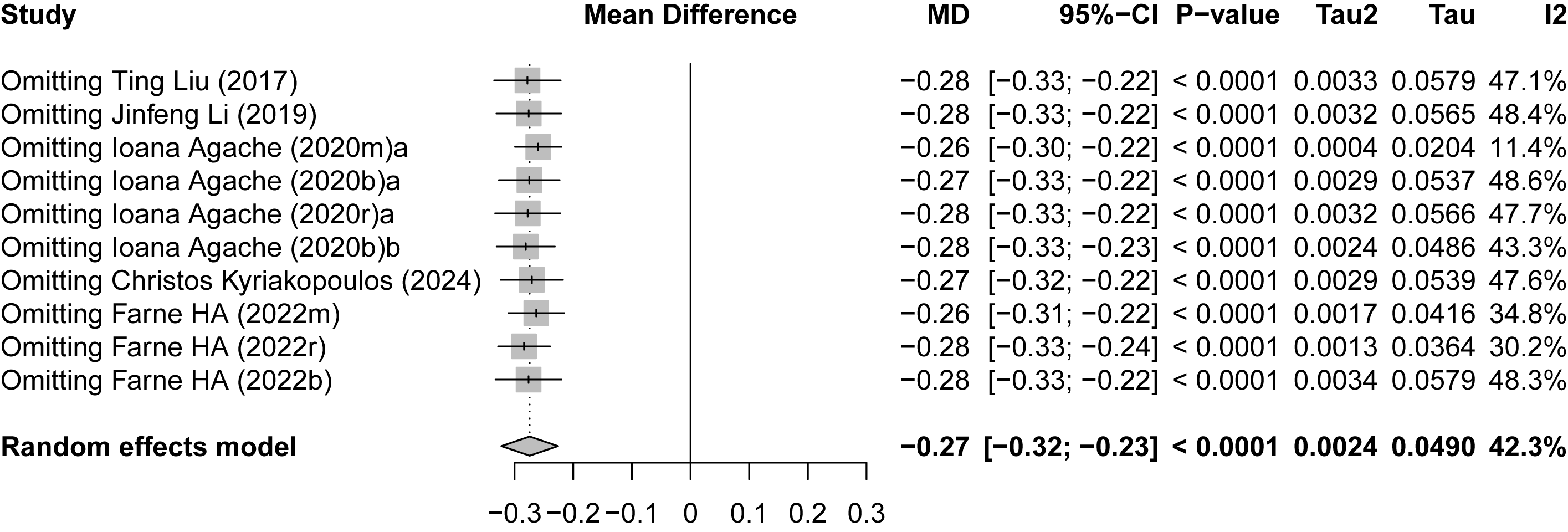


**Supplementary Figure S16.** Sensitivity analysis of mean differences of asthma control questionnaire scores in severe asthma treated with anti-interleukin-5/5Rα treatment.


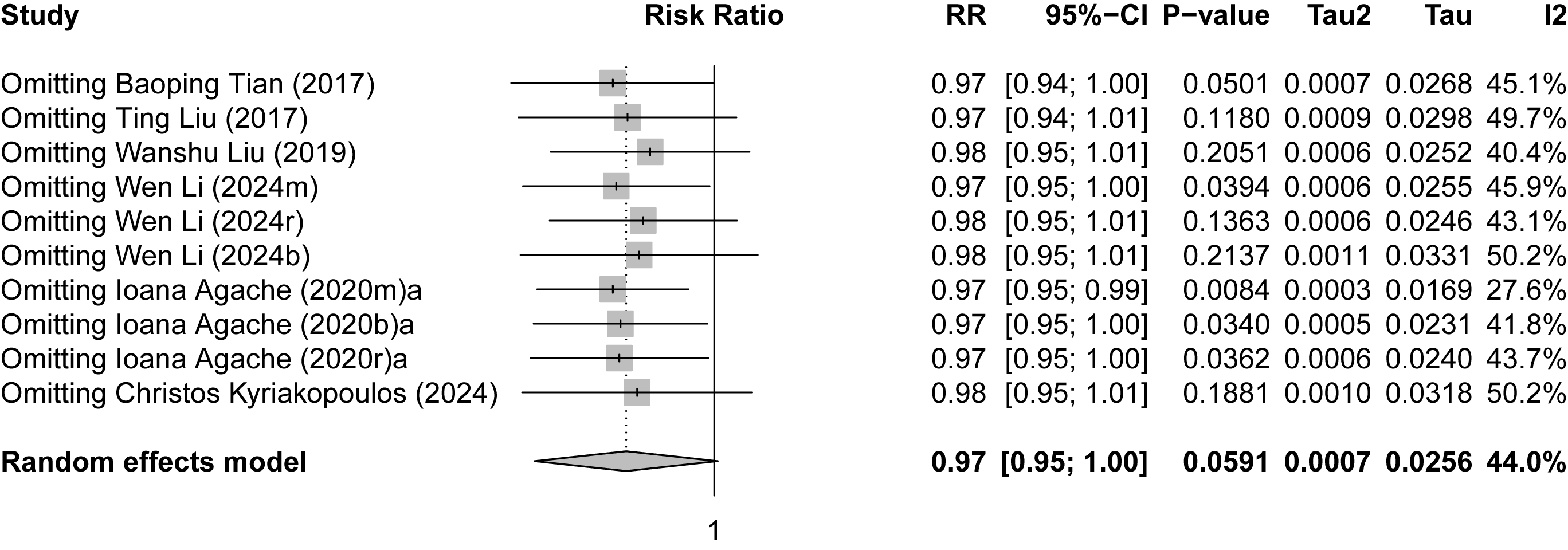


**Supplementary Figure S17.** Sensitivity analysis of risk ratios of adverse events in severe asthma treated with anti-interleukin-5/5Rα treatment.


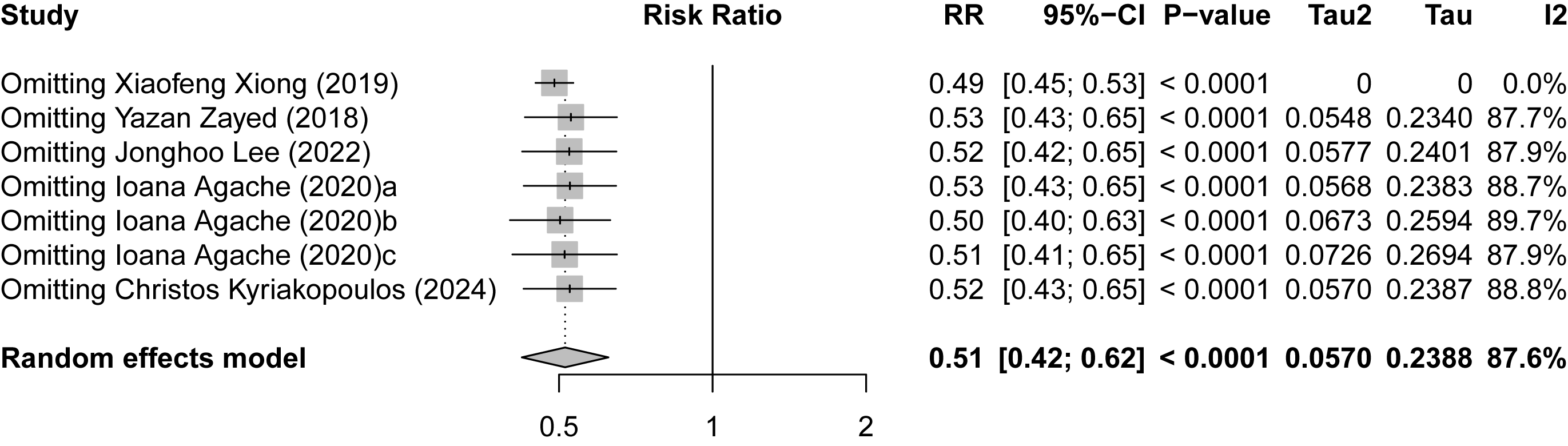


**Supplementary Figure S18.** Sensitivity analysis of risk ratios of asthma exacerbation rate in severe asthma treated with anti-interleukin-4Rα treatment.


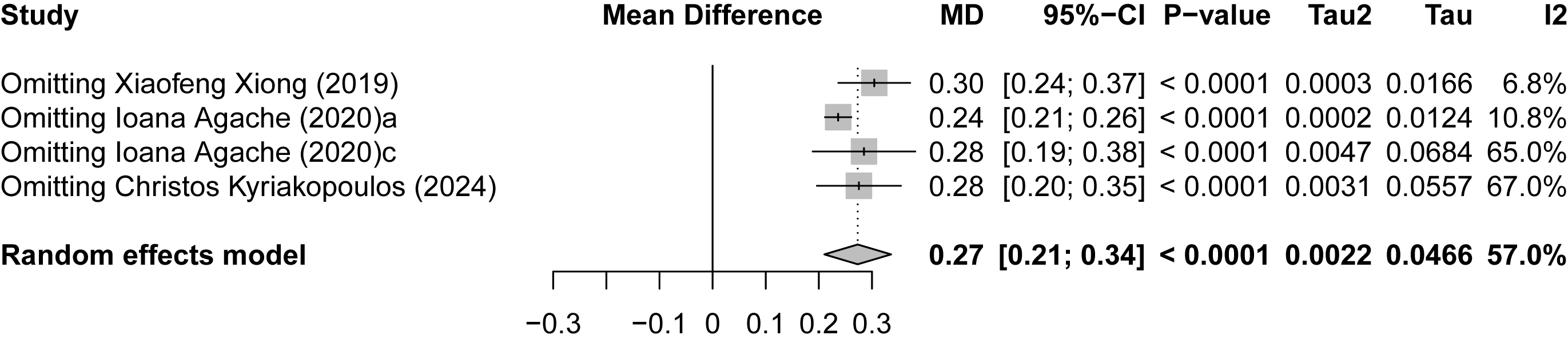


**Supplementary Figure S19.** Sensitivity analysis of mean differences of asthma quality of life questionnaire scores in severe asthma treated with anti-interleukin-4Rα treatment.


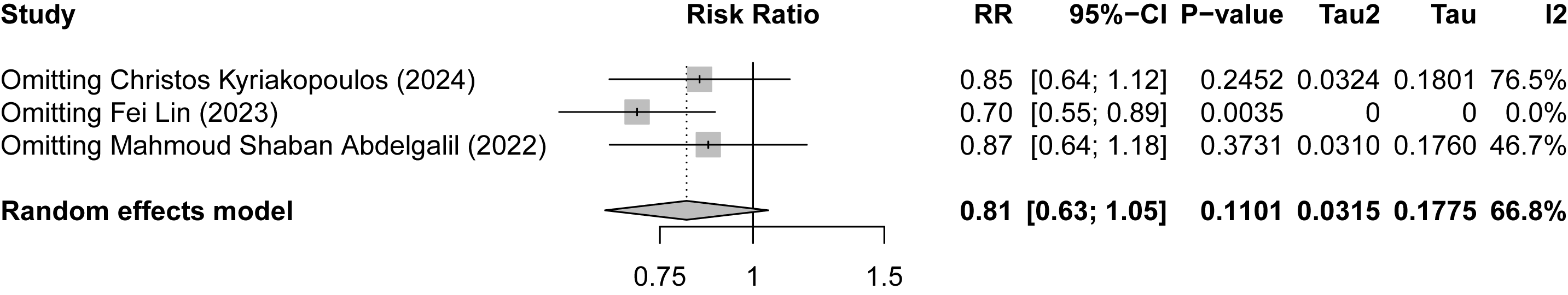


**Supplementary Figure S20.** Sensitivity analysis of risk ratios of adverse events in severe asthma treated with anti-thymic stromal lymphopoietin treatment.


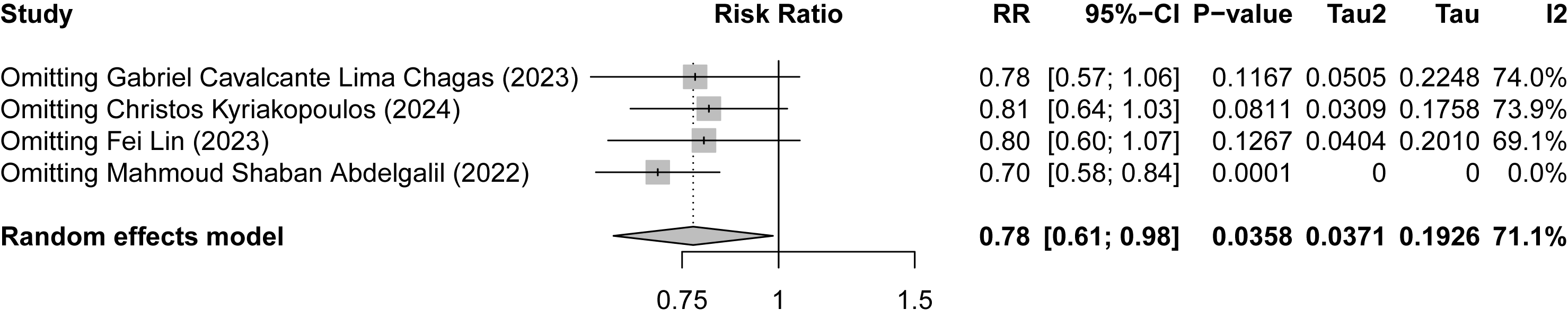


**Supplementary Figure S21.** Sensitivity analysis of risk ratios of serious adverse events in severe asthma treated with anti-thymic stromal lymphopoietin treatment.

## Supplementary Tables

**Supplementary Table S1.** PRISMA 2020 Checklist

| **Section and Topic** | **Item #** | **Checklist item** | **Location where item is reported** |
| --- | --- | --- | --- |
| **TITLE** | | |  |
| Title | 1 | Identify the report as a systematic review. | 1 |
| **ABSTRACT** | | |  |
| Abstract | 2 | See the PRISMA 2020 for Abstracts checklist. | 1 |
| **INTRODUCTION** | | |  |
| Rationale | 3 | Describe the rationale for the review in the context of existing knowledge. | 2 |
| Objectives | 4 | Provide an explicit statement of the objective(s) or question(s) the review addresses. | 2 |
| **METHODS** | | |  |
| Eligibility criteria | 5 | Specify the inclusion and exclusion criteria for the review and how studies were grouped for the syntheses. | 3 |
| Information sources | 6 | Specify all databases, registers, websites, organisations, reference lists and other sources searched or consulted to identify studies. Specify the date when each source was last searched or consulted. | 2 |
| Search strategy | 7 | Present the full search strategies for all databases, registers and websites, including any filters and limits used. | 2 |
| Selection process | 8 | Specify the methods used to decide whether a study met the inclusion criteria of the review, including how many reviewers screened each record and each report retrieved, whether they worked independently, and if applicable, details of automation tools used in the process. | 2-3 |
| Data collection process | 9 | Specify the methods used to collect data from reports, including how many reviewers collected data from each report, whether they worked independently, any processes for obtaining or confirming data from study investigators, and if applicable, details of automation tools used in the process. | 3 |
| Data items | 10a | List and define all outcomes for which data were sought. Specify whether all results that were compatible with each outcome domain in each study were sought (e.g. for all measures, time points, analyses), and if not, the methods used to decide which results to collect. | 3 |
|  | 10b | List and define all other variables for which data were sought (e.g. participant and intervention characteristics, funding sources). Describe any assumptions made about any missing or unclear information. | 3, 9 |
| Study risk of bias assessment | 11 | Specify the methods used to assess risk of bias in the included studies, including details of the tool(s) used, how many reviewers assessed each study and whether they worked independently, and if applicable, details of automation tools used in the process. | NA |
| Effect measures | 12 | Specify for each outcome the effect measure(s) (e.g. risk ratio, mean difference) used in the synthesis or presentation of results. | 3 |
| Synthesis methods | 13a | Describe the processes used to decide which studies were eligible for each synthesis (e.g. tabulating the study intervention characteristics and comparing against the planned groups for each synthesis (item #5)). | 3 |
|  | 13b | Describe any methods required to prepare the data for presentation or synthesis, such as handling of missing summary statistics, or data conversions. | 3 |
|  | 13c | Describe any methods used to tabulate or visually display results of individual studies and syntheses. | 3 |
|  | 13d | Describe any methods used to synthesize results and provide a rationale for the choice(s). If meta-analysis was performed, describe the model(s), method(s) to identify the presence and extent of statistical heterogeneity, and software package(s) used. | 3 |
|  | 13e | Describe any methods used to explore possible causes of heterogeneity among study results (e.g. subgroup analysis, meta-regression). | 3 |
|  | 13f | Describe any sensitivity analyses conducted to assess robustness of the synthesized results. | 4 |
| Reporting bias assessment | 14 | Describe any methods used to assess risk of bias due to missing results in a synthesis (arising from reporting biases). | NA |
| Certainty assessment | 15 | Describe any methods used to assess certainty (or confidence) in the body of evidence for an outcome. | NA |
| **RESULTS** | | |  |
| Study selection | 16a | Describe the results of the search and selection process, from the number of records identified in the search to the number of studies included in the review, ideally using a flow diagram. | 4 |
|  | 16b | Cite studies that might appear to meet the inclusion criteria, but which were excluded, and explain why they were excluded. | 4 |
| Study characteristics | 17 | Cite each included study and present its characteristics. | 4, 13-15 |
| Risk of bias in studies | 18 | Present assessments of risk of bias for each included study. | NA |
| Results of individual studies | 19 | For all outcomes, present, for each study: (a) summary statistics for each group (where appropriate) and (b) an effect estimate and its precision (e.g. confidence/credible interval), ideally using structured tables or plots. | 4-6 |
| Results of syntheses | 20a | For each synthesis, briefly summarise the characteristics and risk of bias among contributing studies. | 4-6 |
|  | 20b | Present results of all statistical syntheses conducted. If meta-analysis was done, present for each the summary estimate and its precision (e.g. confidence/credible interval) and measures of statistical heterogeneity. If comparing groups, describe the direction of the effect. | 4-6 |
|  | 20c | Present results of all investigations of possible causes of heterogeneity among study results. | 4-6 |
|  | 20d | Present results of all sensitivity analyses conducted to assess the robustness of the synthesized results. | 7 |
| Reporting biases | 21 | Present assessments of risk of bias due to missing results (arising from reporting biases) for each synthesis assessed. | NA |
| Certainty of evidence | 22 | Present assessments of certainty (or confidence) in the body of evidence for each outcome assessed. | NA |
| **DISCUSSION** | | |  |
| Discussion | 23a | Provide a general interpretation of the results in the context of other evidence. | 7-8 |
|  | 23b | Discuss any limitations of the evidence included in the review. | 8 |
|  | 23c | Discuss any limitations of the review processes used. | 8 |
|  | 23d | Discuss implications of the results for practice, policy, and future research. | 8 |
| **OTHER INFORMATION** | | |  |
| Registration and protocol | 24a | Provide registration information for the review, including register name and registration number, or state that the review was not registered. | 2 |
|  | 24b | Indicate where the review protocol can be accessed, or state that a protocol was not prepared. | 2 |
|  | 24c | Describe and explain any amendments to information provided at registration or in the protocol. | 2 |
| Support | 25 | Describe sources of financial or non-financial support for the review, and the role of the funders or sponsors in the review. | 9 |
| Competing interests | 26 | Declare any competing interests of review authors. | 9 |
| Availability of data, code and other materials | 27 | Report which of the following are publicly available and where they can be found: template data collection forms; data extracted from included studies; data used for all analyses; analytic code; any other materials used in the review. | 9 |

**Supplementary Table S2.** The details of the search strategy.

| **Pubmed** |
| --- |
| (((((((((((Mepolizumab[MeSH Terms]) OR (Mepolizumab[Title/Abstract]) OR (SB-240563[Title/Abstract])) OR (SB240563[Title/Abstract])) OR (Nucala[Title/Abstract])) OR (Bosatria[Title/Abstract])) OR  ((((((((((Reslizumab[MeSH Terms]) OR (Reslizumab[Title/Abstract]) OR (Cinqair[Title/Abstract])) OR (SCH-55700[Title/Abstract])) OR (SCH 55700[Title/Abstract])) OR (SCH55700[Title/Abstract])) OR (DCP-835[Title/Abstract])) OR (DCP 835[Title/Abstract])) OR (DCP835[Title/Abstract])) OR (CEP-38072[Title/Abstract])) OR (CEP38072[Title/Abstract]))) OR  (((((Benralizumab[MeSH Terms]) OR (Benralizumab[Title/Abstract]) OR (MEDI-563[Title/Abstract])) OR (MEDI 563[Title/Abstract])) OR (Fasenra[Title/Abstract])) OR (BIW-8405[Title/Abstract]))) OR  ((Omalizumab[MeSH Terms]) OR (Xolair[Title/Abstract]))) OR  ((((((Dupilumab[MeSH Terms]) OR (Dupilumab[Title/Abstract]) OR (SAR231893[Title/Abstract])) OR (SAR-231893[Title/Abstract])) OR (REGN668[Title/Abstract])) OR (REGN-668[Title/Abstract])) OR (Dupixent[Title/Abstract]))) OR  (((((((Tezepelumab[MeSH Terms]) OR (Tezepelumab[Title/Abstract]) OR (MEDI-9929[Title/Abstract])) OR (MEDI9929[Title/Abstract])) OR (MEDI-19929[Title/Abstract])) OR (AMG-157[Title/Abstract])) OR (tezspire[Title/Abstract])) OR (tezepelumab-ekko[Title/Abstract]))) AND  ((((Asthma[MeSH Terms]) OR (Asthmas[Title/Abstract])) OR (Asthma, Bronchial[Title/Abstract])) OR (Bronchial Asthma[Title/Abstract]))) AND  ((((((((((((Meta-Analysis as Topic[MeSH Terms]) OR (Meta Analysis[Publication Type])) OR (meta analysis[Title/Abstract])) OR (meta analyses[Title/Abstract])) OR (meta-analysis[Title/Abstract])) OR (meta-analyses[Title/Abstract])) OR (data pooling[Title/Abstract])) OR (data poolings[Title/Abstract])) OR (clinical trial overview[Title/Abstract])) OR (clinical trial overviews[Title/Abstract])) OR (systematic review[Title/Abstract])) OR (systematic reviews[Title/Abstract])) |
| **Embase** |
| #1 'asthma'/exp  #2 'asthma'  #3 'asthma bronchiale' OR 'asthma pulmonale' OR 'asthma, bronchial' OR 'asthmatic' OR 'asthmatic subject' OR 'bronchial asthma' OR 'bronchus asthma' OR 'chronic asthma' OR 'lung allergy'  #4 #1 OR #2 OR #3  #5 'mepolizumab'/exp  #6 'mepolizumab'  #7 'bat 2606' OR 'bat2606' OR 'bosatria' OR 'nucala' OR 'sb 240563' OR 'sb-240563' OR 'sb240563'  #8 'reslizumab'/exp  #9 'reslizumab'  #10 'cep 38072' OR 'cep38072' OR 'cinqaero' OR 'cinqair' OR 'dcp 835' OR 'dcp835' OR 'sch 55700' OR 'sch55700'  #11 'benralizumab'/exp  #12 'benralizumab'  #13 'biw 8405' OR 'biw8405' OR 'fasenra' OR 'khk 4563' OR 'khk4563' OR 'medi 563' OR 'medi563'  #14 'omalizumab'/exp  #15 'omalizumab'  #16 'aomaishu' OR 'cmab 007' OR 'cmab007' OR 'fb 317' OR 'fb317' OR 'gbr 310' OR 'gbr310' OR 'genolair' OR 'gnr 044' OR 'gnr044' OR 'hu 901' OR 'hu901' OR 'monoclonal antibody E 25' OR 'monoclonal antibody E25' OR 'olizumab' OR 'omalizumab alfa' OR 'omalizumab alpha' OR 'omlyclo' OR 'rg 3648' OR 'rg3648' OR 'rhumab 25' OR 'rhumab e25' OR 'sti 004' OR 'sti004' OR 'syn 008' OR 'syn008' OR 'tev 45779' OR 'tev45779' OR 'xolair'  #17 'dupilumab'/exp  #18 'dupilumab'  #19 'bat 2406' OR 'bat2406' OR 'dupixent' OR 'regn 668' OR 'regn668' OR 'sar 231893' OR 'sar231893'  #20 'tezepelumab'/exp  #21 'tezepelumab'  #22 'amg 157' OR 'amg157' OR 'medi 9929' OR 'medi9929' OR 'tezepelumab ekko' OR 'tezepelumab-ekko' OR 'tezspire'  #23 #5 OR #6 OR #7 OR #8 OR #9 OR #10 OR #11 OR #12 OR #13 OR #14 OR #15 OR #16 OR #17 OR #18 OR #19 OR #20 OR #21 OR #22  #24 'systematic review'/exp  #25 'meta analysis'/exp  #26 #24 OR #25  #27 #4 AND #23 AND #26 |
| **Cochrane Library** |
| #1 MeSH descriptor: [Mepolizumab] explode all trees  #2 MeSH descriptor: [Reslizumab] explode all trees  #3 MeSH descriptor: [Benralizumab] explode all trees  #4 MeSH descriptor: [Omalizumab] explode all trees  #5 Xolair  #6 MeSH descriptor: [Dupilumab] explode all trees  #7 MeSH descriptor: [Tezepelumab] explode all trees  #8 #1 OR #2 OR #3 OR #4 OR #5 OR #6 OR #7  #9 MeSH descriptor: [Asthma] explode all trees  #10 Asthma, Bronchial  #11 Asthmas  #12 Bronchial Asthma  #13 #9 OR #10 OR #11 OR #12  #14 MeSH descriptor: [Systematic Review] explode all trees  #15 Review, Systematic  #16 MeSH descriptor: [Meta-Analysis] explode all trees  #17 #14 OR #15 OR #16  #18 #8 AND #13 AND #17 |
| **Web of Science** |
| TS = (Mepolizumab OR Reslizumab OR Benralizumab OR Omalizumab OR Dupilumab OR Tezepelumab) AND TS = (Asthma) AND TS = (Systematic review OR Meta-analysis) |
| **MEDILINE** |
| (SU mepolizumab OR SU reslizumab OR SU benralizumab OR SU omalizumab OR SU dupilumab OR SU tezepelumab) AND (SU asthma) AND (SU systematic review OR SU meta-analysis) |

**Supplementary Table S3.** The list of the excluded studies.

| **Title** | **Exclusion reason** |
| --- | --- |
| Comparison of anti-interleukin-5 therapies in patients with severe asthma: global and indirect meta-analyses of randomized placebo controlled trials | Network meta-analysis |
| Efficacy and safety of anti-interleukin-5 therapy in patients with asthma: A pairwise and Bayesian network meta-analysis | Network meta-analysis |
| The effect of treatment with omalizumab, an anti-IgE antibody, on asthma exacerbations and emergency medical visits in patients with severe persistent asthma | No available data |
| Response of Older Patients with IgE-Mediated Asthma to Omalizumab: A Pooled Analysis | No available data |
| Use of dupilumab on the treatment of moderate-to-severe asthma: a systematic review | No available data |
| Omalizumab for the treatment of severe persistent allergic asthma: a systematic review and economic evaluation | No available data |
| Impact of omalizumab on quality-of-life outcomes in patients with moderate-to-severe allergic asthma | No available data |
| Patient-reported outcomes in moderate-to-severe allergic asthmatics treated with omalizumab: a systematic literature review of randomized controlled trials | No available data |
| Real-world Effectiveness of Mepolizumab in Severe Eosinophilic Asthma: A Systematic Review and Meta-analysis | SR/MA of non-RCTs |
| ‘Real-world’ effectiveness of omalizumab in adults with severe allergic asthma: a meta-analysis | SR/MA of non-RCTs |
| Real-World Effectiveness of Omalizumab in Severe Allergic Asthma: A Meta-Analysis of Observational Studies | SR/MA of non-RCTs |
| Mepolizumab improves clinical outcomes in patients with severe asthma and comorbid conditions | No available data |
| Mepolizumab reduces exacerbations in patients with severe eosinophilic asthma, irrespective of body weight/body mass index: meta-analysis of MENSA and MUSCA | No available data |
| “Real-life” Effectiveness Studies of Omalizumab in Adult Patients with Severe Allergic Asthma: Meta-analysis | SR/MA of non-RCTs |
| Real-world efficacy of treatment with benralizumab, dupilumab, mepolizumab and reslizumab for severe asthma: A systematic review and meta-analysis | SR/MA of non-RCTs |
| Short- and long-term real-world effectiveness of omalizumab in severe allergic asthma: systematic review of 42 studies published 2008-2018 | SR/MA of non-RCTs |
| Effectiveness and Safety Studies of Omalizumab in Children and Adolescents With Moderate-To-Severe Asthma | No available data |
| Clinical effects of mepolizumab in patients with severe eosinophilic asthma according to background therapy: A meta-analysis | No available data |
| Efficacy of Anti-Interleukin-5 Therapy with Mepolizumab in Patients with Asthma: A Meta-Analysis of Randomized Placebo-Controlled Trials | No available data |
| Efficacy and Safety of Anti-Interleukin-5 Therapy in Patients with Asthma: A Systematic Review and Meta-Analysis | No available data |
| Matching-adjusted comparison of oral corticosteroid reduction in asthma: Systematic review of biologics | Network meta-analysis |
| Tezepelumab compared with other biologics for the treatment of severe asthma: a systematic review and indirect treatment comparison | Network meta-analysis |
| Anti–IL-5 treatments in patients with severe asthma by blood eosinophil thresholds: Indirect treatment comparison | Network meta-analysis |
| Comparative efficacy of mepolizumab, benralizumab, and dupilumab in eosinophilic asthma: A Bayesian network meta-analysis | Network meta-analysis |
| Comparative Efficacy of Tezepelumab to Mepolizumab, Benralizumab, and Dupilumab in Eosinophilic Asthma: A Bayesian Network Meta-analysis | Network meta-analysis |
| Comparative Efficacy of Anti IL-4, IL-5 and IL-13 Drugs for Treatment of Eosinophilic Asthma: A Network Meta-analysis | Network meta-analysis |
| Monoclonal antibodies in type 2 asthma: a systematic review and network meta-analysis | Network meta-analysis |
| Effect of Anti-IL5, Anti-IL5R, Anti-IL13 Therapy on Asthma Exacerbations: A Network Meta-analysis | Network meta-analysis |
| Omalizumab versus Mepolizumab as add-on therapy in asthma patients not well controlled on at least an inhaled corticosteroid: A network meta-analysis | Network meta-analysis |
| A comparison of the effectiveness of biologic therapies for asthma A systematic review and network meta-analysis | Network meta-analysis |
| Monoclonal antibodies in severe asthma: is it worth it? | No available data |
| Improvement in quality of life with omalizumab in patients with severe allergic asthma | No available data |
| Mepolizumab improves work productivity, activity limitation, symptoms, and rescue medication use in severe eosinophilic asthma | No available data |
| Effect of mepolizumab in severe eosinophilic asthma according to omalizumab eligibility | No available data |
| Reslizumab in patients with inadequately controlled late-onset asthma and elevated blood eosinophils | No available data |
| Effect of omalizumab on lung function and eosinophil levels in adolescents with moderate-to-severe allergic asthma | No available data |

**Supplementary Table S4.** Methodological quality assessment of included studies using AMSTAR 2.

| Author (Year of publication) | Item 1 | Item 2 | Item 3 | Item 4 | Item 5 | Item 6 | Item 7 | Item 8 | Item 9 | Item 10 | Item 11 | Item 12 | Item 13 | Item 14 | Item 15 | Item 16 | Overall rating |
| --- | --- | --- | --- | --- | --- | --- | --- | --- | --- | --- | --- | --- | --- | --- | --- | --- | --- |
| Baoping Tian (2017) | 1 | 1 | 1 | 1 | 1 | 1 | 0 | 1 | 1 | 0 | 1 | 1 | 1 | 1 | 1 | 1 | Low |
| Ting Liu (2017) | 1 | 1 | 1 | 1 | 1 | 1 | 0 | 1 | 1 | 0 | 1 | 1 | 1 | 1 | 1 | 0 | Low |
| Wanshu Liu (2019) | 1 | 1 | 1 | 1 | 1 | 1 | 0 | 1 | 1 | 1 | 1 | 1 | 1 | 1 | 1 | 0 | Low |
| Xiaofeng Xiong (2019) | 1 | 1 | 1 | 1 | 1 | 1 | 0 | 1 | 1 | 1 | 1 | 1 | 1 | 1 | 1 | 1 | Low |
| Yazan Zayed (2018) | 1 | 1 | 1 | 1 | 1 | 1 | 0 | 1 | 1 | 0 | 1 | 1 | 1 | 1 | 1 | 1 | Low |
| Wen Li (2024) | 1 | 1 | 1 | 1 | 1 | 0 | 0 | 1 | 1 | 0 | 1 | 1 | 1 | 1 | 1 | 1 | Low |
| Steven W. Yancey (2017) | 1 | 1 | 1 | 1 | 0 | 0 | 0 | 1 | 1 | 1 | 1 | 1 | 1 | 1 | 1 | 1 | Low |
| Junyi Liao (2024) | 1 | 1 | 1 | 1 | 1 | 1 | 0 | 1 | 1 | 0 | 1 | 1 | 1 | 1 | 0 | 1 | Critically low |
| Zhuo Fu (2020) | 1 | 1 | 1 | 1 | 0 | 1 | 0 | 1 | 1 | 0 | 1 | 1 | 1 | 1 | 1 | 1 | Low |
| Tianwen Lai (2015) | 1 | 1 | 1 | 1 | 0 | 1 | 1 | 1 | 1 | 0 | 1 | 1 | 1 | 1 | 1 | 1 | Moderate |
| Dandan Lang (2023) | 1 | 1 | 1 | 1 | 1 | 1 | 0 | 1 | 1 | 0 | 1 | 1 | 1 | 1 | 1 | 1 | Low |
| Gustavo J. Rodrigo (2015) | 1 | 1 | 1 | 1 | 1 | 1 | 0 | 1 | 1 | 0 | 1 | 1 | 1 | 1 | 0 | 1 | Critically low |
| Jinfeng Li (2019) | 1 | 1 | 1 | 1 | 0 | 1 | 0 | 1 | 1 | 0 | 1 | 1 | 1 | 1 | 1 | 0 | Low |
| Jonghoo Lee (2022) | 1 | 1 | 1 | 1 | 1 | 1 | 0 | 1 | 1 | 0 | 1 | 1 | 1 | 1 | 1 | 1 | Low |
| Ioana Agache (2020)a | 1 | 1 | 1 | 1 | 1 | 1 | 1 | 1 | 1 | 1 | 1 | 1 | 1 | 1 | 1 | 1 | High |
| Ioana Agache (2020)b | 1 | 1 | 1 | 1 | 1 | 1 | 1 | 1 | 1 | 1 | 1 | 1 | 1 | 1 | 1 | 1 | High |
| Ioana Agache (2020)c | 1 | 1 | 1 | 1 | 1 | 1 | 1 | 1 | 1 | 1 | 1 | 1 | 1 | 1 | 1 | 1 | High |
| Gabriel Cavalcante Lima Chagas (2023) | 1 | 1 | 1 | 1 | 1 | 1 | 0 | 1 | 1 | 0 | 1 | 1 | 1 | 1 | 0 | 0 | Critically low |
| Christos Kyriakopoulos (2024) | 1 | 1 | 1 | 1 | 1 | 1 | 0 | 1 | 1 | 0 | 1 | 1 | 1 | 1 | 1 | 1 | Low |
| Fei Lin (2023) | 1 | 1 | 1 | 1 | 1 | 1 | 0 | 1 | 1 | 1 | 1 | 1 | 1 | 1 | 0 | 0 | Critically low |
| Mahmoud Shaban Abdelgalil (2022) | 1 | 1 | 1 | 1 | 1 | 1 | 0 | 1 | 1 | 0 | 1 | 1 | 1 | 1 | 1 | 1 | Low |
| Normansell R (2014) | 1 | 1 | 1 | 1 | 1 | 1 | 1 | 1 | 1 | 0 | 1 | 1 | 1 | 1 | 1 | 1 | High |
| Farne HA (2022) | 1 | 1 | 1 | 1 | 1 | 1 | 1 | 1 | 1 | 1 | 1 | 1 | 1 | 1 | 1 | 1 | High |

Note: AMSTAR 2 used sixteen items to assess methodological quality of systematic reviews on the basis of the validity of review design, literature screening, data extraction, and individual study quality assessment. Among these items, AMSTAR 2 designated seven “critical domains” that can critically affect the validity of a review (e.g., items 2, 4, 7, 9, 11, 13, and 15). Meta-analyses were considered as high quality if they met all “critical domains” with other items satisfied ≥8, and other meta-analyses that met all “critical domains” were considered as moderate quality. Besides, meta-analyses with one unsatisfied critical domain were assigned to low quality, and meta-analyses with more than one unsatisfied critical domain were considered as critically low quality.

The items are as follows:

Item 1: Did the research questions and inclusion criteria for the review include the components of PICO?

Item 2: Did the report of the review contain an explicit statement that the review methods were established prior to conduct of the review and did the report justify any significant deviations from the protocol?

Item 3: Did the review authors explain their selection of the study designs for inclusion in the review?

Item 4: Did the review authors use a comprehensive literature search strategy?

Item 5: Did the review authors perform study selection in duplicate?

Item 6: Did the review authors perform data extraction in duplicate?

Item 7: Did the review authors provide a list of excluded studies and justify the exclusions?

Item 8: Did the review authors describe the included studies in adequate detail?

Item 9: Did the review authors use a satisfactory technique for assessing the risk of bias (RoB) in individual studies that were included in the review?

Item 10: Did the review authors report on the sources of funding for the studies included in the review?

Item 11: If meta-analysis was justified, did the review authors use appropriate methods for statistical combination of results?

Item 12: If meta-analysis was performed, did the review authors assess the potential impact of RoB in individual studies on the results of the meta-analysis or other evidence synthesis?

Item 13: Did the review authors account for RoB in individual studies when interpreting/discussing the results of the review?

Item 14: Did the review authors provide a satisfactory explanation for, and discussion of, any heterogeneity observed in the results of the review? Item 15: If they performed quantitative synthesis, did the review authors carry out an adequate investigation of publication bias (small-study bias) and discuss its likely impact on the results of the review?

Item 16: Did the review authors report any potential sources of conflict of interest, including any funding they received for conducting the review?
